# Functional and structural characteristics of HLA-B*13:01-mediated specific T cells reaction in dapsone-induced drug hypersensitivity

**DOI:** 10.1186/s12929-022-00845-8

**Published:** 2022-08-13

**Authors:** Haiqin Jiang, Chuang-Wei Wang, Zhaoxi Wang, Yufei Dai, Yanping Zhu, Yun-Shien Lee, Yang Cao, Wen-Hung Chung, Songying Ouyang, Hongsheng Wang

**Affiliations:** 1 Department of Mycobacterium, Jiangsu Key Laboratory of Molecular Biology for Skin Diseases and STIs, Institute of Dermatology & Hospital for Skin Diseases, Chinese Academy of Medical Sciences & Peking Union Medical College, Nanjing, China; 2 Centre for Global Health, School of Public Health, Nanjing Medical University, Nanjing, China; 3 Department of Dermatology, Drug Hypersensitivity Clinical and Research Center, Cancer Vaccine and Immune Cell Therapy Core Laboratory, Chang Gung Memorial Hospital, Taipei and Keelung, Linkou, Taiwan; 4 Chang Gung Immunology Consortium, Chang Gung Memorial Hospital and Chang Gung University, Taoyuan, Taiwan; 5 Department of Dermatology, Xiamen Chang Gung Hospital, Xiamen, China; 6 The Key Laboratory of Innate Immune Biology of Fujian Province, Biomedical Research Center of South China, Key Laboratory of OptoElectronic Science and Technology for Medicine of Ministry of Education, College of Life Sciences, Fujian Normal University, Fujian, China; 7 National Institute of Occupational Health and Poison Control, Chinese Center for Disease Control and Prevention, Beijing, China; 8 National Laboratory of BiomacromoleculesInstitute of Biophysics, Chinese Academy of Sciences, Beijing, China; 9 Department of Biotechnology, Ming Chuan University, Taoyuan, Taiwan; 10 College of Life Sciences, Sichuan University, Chengdu, China; 11 College of Medicine, Chang Gung University, Taoyuan, Taiwan; 12 Immune-Oncology Center of Excellence, Chang Gung Memorial Hospital, Linkou, Taiwan; 13 Department of Dermatology, Beijing Tsinghua Chang Gung Hospital, School of Clinical Medicine, Tsinghua University, Beijing, China; 14 School of Medicine, Shanghai Jiao Tong University, Shanghai, China; 15 Genomic Medicine Core Laboratory, Chang Gung Memorial Hospital, Linkou, Taiwan

**Keywords:** Dapsone, Dapsone-induced hypersensitivity syndrome, HLA-B*13:01, T-cell receptor

## Abstract

**Background:**

Severe cutaneous adverse drug reactions (SCARs) are a group of serious clinical conditions caused by immune reaction to certain drugs. The allelic variance of human leukocyte antigens of HLA-B*13:01 has been strongly associated with hypersensitivities induced by dapsone (DDS). T-cell receptor mediated activation of cytotoxic T lymphocytes (CTLs) has also been suggested to play an essential role in pathogenesis of SCARs. However, HLA-B*13:01-DDS-TCR immune synapse that plays role in drug-induced hypersensitivity syndrome (DIHS) associated T cells activation remains uncharacterized.

**Methods:**

To investigate the molecular mechanisms for HLA-B*13:01 in the pathogenesis of Dapsone-induced drug hypersensitivity (DDS-DIHS), we performed crystallization and expanded drug-specific CTLs to analyze the pathological role of DDS-DIHS.

**Results:**

Results showed the crystal structure of HLA-B*13:01-beta-2-microglobulin (β2M) complex at 1.5 Å resolution and performed mutation assays demonstrating that I118 or I119, and R121 of HLA-B*13:01 were the key residues that mediate the binding of DDS. Subsequent single-cell TCR and RNA sequencing indicated that TCRs composed of paired TRAV12-3/TRBV28 clonotype with shared CDR3 region specifically recognize HLA-B*13:01-DDS complex to trigger inflammatory cytokines associated with DDS-DIHS.

**Conclusion:**

Our study identified the novel p-i-HLA/TCR as the model of interaction between HLA-B*13:01, DDS and the clonotype-specific TCR in DDS-DIHS.

**Graphical Abstract:**

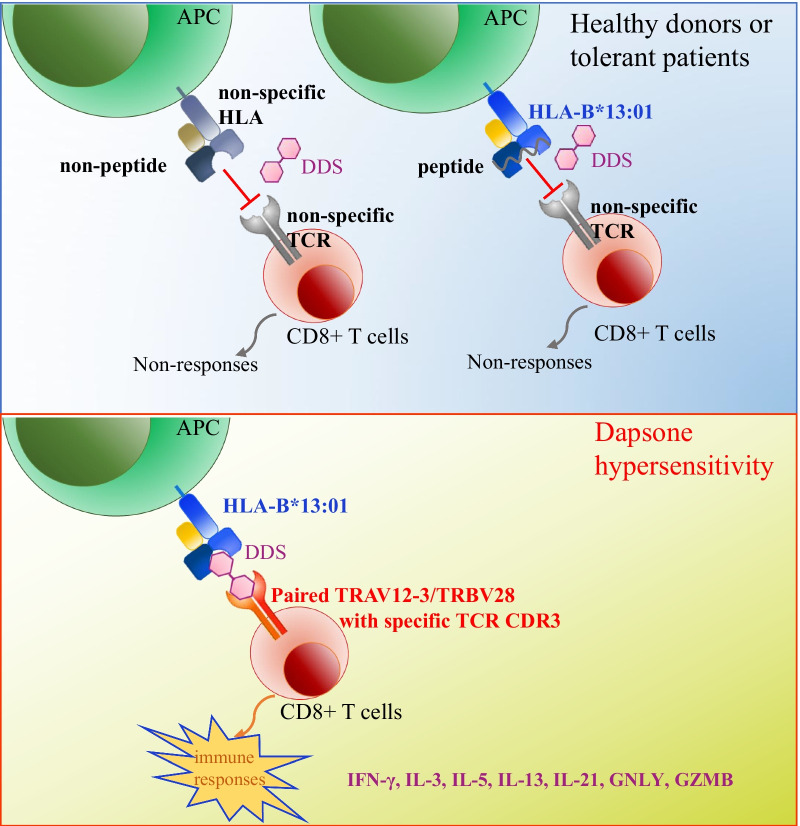

**Supplementary Information:**

The online version contains supplementary material available at 10.1186/s12929-022-00845-8.

## Background

Severe cutaneous adverse drug reactions (SCARs), including drug-induced hypersensitivity syndrome or drug reaction with eosinophilia and systemic symptoms (DRESS), Stevens-Johnson syndrome (SJS) and toxic epidermal necrolysis (TEN), are a group of potentially life-threatening adverse drug reactions with different clinical presentations and pathogenesis [1, 2]. SCARs are delayed-type hypersensitivity reactions (i.e., drug-induced immune reactions mediated by T cells that occur after a minimum of 3–4 days of exposure to the causative drug). Naive T cells are primed upon initial treatment with the causative drug, following which a pool of memory T cells is gradually formed by re-stimulation during continued exposure. Accordingly, SCARs resolve when treatment with the causative drug is discontinued and develop more rapidly upon renewal of its administration [3, 4].

Individuals with predisposition to develop SCARs appear to possess specific human leukocyte antigen (HLA) alleles, which have been found to trigger the condition by mediating drug-induced T cell activation [5–9]. Drug-specific T-cell receptors (TCRs) have been observed in oligoclonal T cell populations isolated from skin lesions and blood samples of allopurinol-induced SCAR patients with HLA-B*58:01 allele [10] and carbamazepine-induced SJS/TEN patients with HLA-B*15:02 allele [11]. Several theories, such as hapten (and pro-hapten), p-i (pharmacological interaction with immune receptors) [12, 13], peptide repertoire alteration [14] and altered TCR repertoire model, have been proposed to explain the HLA-associated drug hypersensitivity [15, 16]. More recently, a unique population of T cells, termed skin-resident memory T cells, have been identified as possible SCAR mediators [10, 16]. In addition, high-resolution crystal structures of self-peptides presented by abacavir-modified HLA-B*57:01 have been reported, providing valuable insight into HLA-associated drug hypersensitivity on molecular level [11, 17]. Besides, the structural bioinformatics approach, which has seen remarkable advance in recent years, is a promising way to computationally elucidate the structural basis, dynamics and energetic properties of HLA-drug association as well as its pathological role in SCARs.

The HLA-B*13:01 allele has been identified as an important risk allele for SCARs induced by a number of drugs, including dapsone (diaminodiphenyl sulfone, DDS) [18–24]. DDS-induced hypersensitivity syndrome (DDS-DIHS) is one of the major causes of mortality amongst leprosy patients possessing the HLA-B*13:01 allele (i.e., about 2% of leprosy patients with DDS therapy, mortality rate 12.5%)[25–27]. DDS has also been used to treat various dermatologic and non-dermatologic diseases, including *Pneumocystis jirovecii* pneumonia [28], dermatitis herpetiformis [29], immune thrombocytopaenia [30] and eosinophilic fasciitis [31]. Previous studies found that DDS-specific cytotoxic T cells were significantly activated when co-cultured with HLA-B*13:01-expressing antigen presenting cells (APCs) in the presence of DDS [32, 33]. However, HLA-B*13:01-DDS-TCR immune synapse that plays role in DDS-DIHS associated T cells activation remains uncharacterized, which makes prevention and treatment efforts more difficult.

In this study, we used structural biology, bioinformatics and mutation assays to demonstrate that extra-protein crystallization of HLA-B*13:01 and DDS binds directly to HLA-B*13:01 and to determine key HLA-B*13:01 residues mediating the binding. These key residues also explain why DDS-DIHS is specifically associated with HLA-B*13:01 and not the highly homologous HLA-B*13:02. Moreover, we carried out RNA-sequencing and single-cell TCR sequencing to identify DDS-specific TCR clonotypes from the blister cells and peripheral blood mononuclear cells (PBMCs) of DDS-DIHS and DDS-SJS patients. Using in silico structure modeling, we show that DDS interacts directly both with HLA-B*13:01 and the DDS-specific TCR, indicating that DIHS-associated TCL activation is mediated through the p-i -mediated drug interaction with HLA and TCR (p-i HLA/TCR model). The results of this study provide new insights for prevention and treatment of DDS-DIHS, and highlight the p-i HLA/TCR model as one of possible pathomechanisms for HLA-associated drug hypersensitivities.

## Materials and methods

### Study design and patient recruitment

From 2012 to 2020, we collected twelve clinical information, as well as eleven blood cells samples of DRESS and one blister cells sample of SJS/TEN from patients diagnosed with DDS-induced hypersensitivity reactions (DDS-DIHS), at the Institute of Dermatology, Chinese Academy of Medical Sciences, Nanjing and the Chang Gung Memorial Hospitals, Taiwan. DRESS was diagnosed according to the RegiSCAR criteria [34, 35] and was characterized by extensive erythematous maculopapular rash with periorbital edema and desquamation. The diagnosis of DRESS using the criteria and scoring system of the RegiSCAR group include cutaneous involvement with typical skin eruptions (e.g., exfoliative dermatitis, generalized maculopapular exanthema), fever (≧ 38.5 °C), enlarged lymph nodes (≧ 2 sites, ≧ 1 cm), presence of atypical lymphocytes and eosinophilia, systemic involvement (e.g., liver, kidney, and lung), time of resolution, and the evaluation of other potential causes. The clinical course, dosage and duration of dapsone, systemic involvement, and mortality were analyzed. The drug causality of SJS and DIHS was determined by the algorithm of drug causality for epidermal necrolysis (ALDEN) and Naranjo algorithm following the guidelines for assessment of drug causality published by the RegiSCAR group [35–38]. Only the cases with probable or certain causal association with DDS (ALDEN score greater than 4 or Naranjo algorithm greater than 5 were recruited. All DDS-DIHS patients and one DDS-SJS patient were HLA-B*13:01-positive, and were assessed by two dermatologists through review of photographs, pathology tissue slides, and medical records. We also recruited ten DDS-tolerant control subjects who had been administered DDS for at least 3 months without any cutaneous adverse reactions. Among them, three were HLA-B*13:01-positive, and seven were HLA-B*13:01-negative leprosy patient (see Table 1). In addition, twelve healthy control individuals were enrolled. Approvals were obtained from the institutional review board (IRB No. 100-4657A3, 201601761B0, 201902171A3 and 202001645B0), and informed consent was obtained from each participant.

**Table 1:** Clinical characteristics of twelve patients and ten tolerant controls with dapsone-induced hypersensitivity reactions

*Characteristic*	Eleven DDS-DIHS and one DDS-SJS patients	Tolerant patient (n = 10)
Age, y (mean ± SD)	43.1 ± 17.0	46.7 ± 13.5
Sex, M:F	5:7	5:5
Fever ≥ 38.5 °C, n (%)	12 (100%)	0 (0%)
Typical skin eruptions, n (%)		
Exfoliative dermatitis	2 (16.7%)	
Erythrodema	10 (83.3%)	
Enlarged lymph nodes, n (%)		
≥ 2 sites	12 (100%)	
≥ 1 cm		
Eosinophilia, n (%)		
Grade 2 (≥ 1500/µL)	6 (50%)	
Grade 1 (700–1499/µL)	6 (50%)	
Mucosal involvement, n (%)		
Oral	2 (16.7%)	
Conjunctival	2 (16.7%)	
Genital	2 (16.7%)	
Internal organ involvement, n (%)		
Liver	12 (100%)	
Kidney	3 (25.0%)	
EBV/HHV6 reactivation*, n (%)		
None	7 (58.3%)	
n.a.	5 (41.7%)	
HLA-B*13:01 positive	12 (100%)	3 (30%)
DIHS/DRESS score*		
6	3 (25%)	
5	3 (25%)	
4	6 (50%)	

*Calculated according to RegiSCAR group diagnosis score for DIHS/DRESS (http://www.regiscar.org/index.html). n.a., not available

### Chemicals, vectors and cell lines

DDS, the DDS metabolite N-acetyl DDS (NAD), sulfadiazine and trichloroethylene were purchased from Sigma-Aldrich (St Louis, Mo). EBV-transformed autologous B-cell lines (B-LCLs), and HLA class I-deficient lymphoblastoid B-cell line (Hmy2.C1R), which were used as antigen-presenting cells (APC), were obtained from ATCC (Maryland, USA). For mutation assays, full-length open-reading frames encoding HLA-B*13:01, HLA-B*13:02 and β2M cloned into VP64-GFP vector. The vectors encoding wild-type HLA-B*13:01 and HLA-B*13:02 were then used as templates for generation of single-site and two-site HLA-B*13:01 and HLA-B*13:02 mutant variants with site-directed mutagenesis (Table 2). The VP64-GFP vectors encoding wild-type or one of mutant HLA-B*13:01 or HLA-B*13:02 variants were then transfected along with VP64-GFP-β2M, pspax2 and pMD2.G to Hmy2. C1R cells (HLA-B-deficient cells) and sorted by flow cytometry with GFP fluorescent protein as a reporter. Stable expression of the C1R transmembrane HLA-B*13:01 (C1R-HLA-B*13:01) clone was determined using flow cytometry by determining protein expression with anti-HLA antibody (w6/32, eBioscience).

**Table 2:** X-ray crystallography data collection and refinement statistics

Dataset	HLA-B*13:01-β2M-peptide
Data collection	
Beamline	BL-17U1, SSRF
Wavelength (Å)	0.9792
Resolution range (Å)*	50.00–1.50 (1.53–1.50)
Space group	*P*212121
Cell dimensions	
a, b, c (Å)	50.30, 82.77, 110.44
α, β, γ (°)	90.00, 90.00, 90.00
Total reflections	2,150,046 (74,696)
Unique reflections	2,142,023 (74,568)
Multiplicity	12.5
Completeness (%)	99.9 (98.2)
* Mean I/sigma(I)*	44.2 (3.5)
* R*-meas	0.100 (0.412)
* R*-pim	0.028 (0.146)
CC1/2	0.995 (0.932)
Refinement	
Reflections used in refinement	74,599
Reflections used for R-free	3761
* Rwork*	0.175
* Rfree*	0.195
Number of atoms	3807
Protein	3164
Solvent	643
RMS (bonds)	0.006
RMS (angles)	1.106
Ramachandran favored (%)	98.68
Ramachandran allowed (%)	1.06
Ramachandran outliers (%)	0.26

*For each structure one crystal was used

*Values in parentheses are for highest-resolution shell

### Expression and purification of recombinant proteins for crystallographic experiments

Gene sequences for HLA-B*13:01 and beta-2-microglobulin (GenBank ID: AAA59627.1 and AAA51811.1) were synthesized at Sangon Biotech (China). The genes were cloned into the pET21a vector with an C-terminal 6 × His tag and expressed in *Escherichia coli* BL21 (DE3) cells. The cells were grown in LB medium at 37 °C until the OD_600_ nm reached 0.6, after which the expression of the recombinant proteins was induced at 16 °C for 20 h with the final concentration of isopropyl-B-D-1-thiogalactopyranoside (IPTG) of 0.1 mM. The cells were then harvested by centrifugation at 12,000 × g for 20 min, resuspended in lysis buffer containing 6 M guanidine-HCl and ultrasonicated. Recombinant proteins (180 mg HLA-B*13:01 and 60 mg β2M) were renatured overnight at 4 °C in dialysis buffer (0.1 M Tris pH = 8.0, 2 mM EDTA, 400 mM l-arginine-HCl, 0.5 mM oxidized glutathione), following which the precipitate was removed by centrifugation. The clear supernatant was loaded onto a 5 mL nickel-nitrilotriacetic acid (Ni–NTA) resin gravity column (Qiagen) pre-equilibrated with binding buffer (20 mM Tris–HCl pH = 8.0, 150 mM NaCl). The column was successively washed with 50 mL binding buffer containing 20 mM imidazole and 100 mM imidazole, and target protein was eluted using binding buffer containing 500 mM imidazole. The target proteins were then concentrated and loaded onto a Superdex G200 size-exclusion chromatography (SEC) column (120 mL, GE Healthcare Life Sciences, USA) pre-equilibrated with SEC buffer (20 mM Tris–HCl pH 8.0, 150 mM NaCl and 2 mM DTT). As initial SEC results indicated that the majority of HLA-B*13:01 did not bind to β2M, we used different oligopeptides predicted by NetMHC 4.0 Server (http://www.cbs.dtu.dk/services/NetMHC/) to stabilize the HLA-B*13:01-β2M complex. After purification of HLA-B*13:01 and β2M, the predicted peptides were added respectively. The formation of HLA-B*13:01-β2M complex was verified by SEC combined with SDS-PAGE, and finally found that the peptide (RQDILDLWI) could promote the formation of the complex.

### Crystallization

Initial crystallization screens for the HLA-B*13:01-β2M-peptide complex (concentrated to 5–7 µg/mL) were performed with commercial crystallization screening kits using the sitting-drop vapor diffusion method at 16 °C. Crystallization drops contained 0.5 μL of the protein complex solution mixed with 0.5 μL of reservoir solution. Diffraction quality crystals were grown in 0.1 M HEPES (pH 6.5–7.5), 20% (v/v) PEG 4000, and 0.2 M NaCl. All crystals were cryoprotected in the reservoir solution supplemented with 20–25% (v/v) glycerol and flash-frozen in liquid nitrogen.

### Data collection and structure determination

X-ray diffraction data for the HLA-B*13:01-β2M-peptide complex crystal was collected at the beamline BL-17U1 of the Shanghai Synchrotron Radiation Facility (SSRF). All data were indexed and scaled using HKL2000 software. The structure of HLA-B*13:01-β2M-peptide complex was determined by molecular replacement with MOLREP in PHENIX [39] using the structure of HLA-B*44:03 in complex with Epstein-Barr virus BZLF1-derived peptide (PDB ID: 4JQX) as the search model. AutoBuild in PHENIX was used to automatically build the structure model. After several rounds of positional and B-factor refinement using Phenix. Refine with TLS parameters alternated by manual model revision in Coot [40], model quality was verified using the PROCHECK program (https://www.ebi.ac.uk/thornton-srv/software/PROCHECK). The quality of the final model was validated with MolProbity [41]. Structures were analyzed with the PDBePISA (Protein Interfaces, Surfaces, and Assemblies) tool on Dali server (http://ekhidna2.biocenter.helsinki.fi/dali). Detailed information on data collection and refinement statistics is listed in the Table 2. All structural figures were prepared in PyMOL (http://www.pymol.org).

### Surface plasmon resonance

Biacore T200 surface plasmon resonance (SPR) system (GE Healthcare, Piscataway, NJ) was used to analyze the interaction between HLA-B*13:01 and different drugs. The recombinant HLA-B*13:01 and β2-microglobulin (Chemicon International, Temecula, Calif) were incubated together in PBS buffer and then immobilized on the chips via amine coupling reaction. The drugs used for the analysis were dissolved in PBS supplemented with 5% dimethyl sulfoxide (DMSO and flowed through the solid phase. Responses of the interaction were reference subtracted and corrected with a standard curve for the DMSO effects. BIA Evaluation software Version 1.0 was used for data analysis. The Kd value were determined by three independent experiments.

### HLA-B*13:01 fix and pulse and binding assay

We investigated the DDS/HLA-B*13:01 interaction based on cytotoxic T lymphocytes (CTLs) activity. Specific CTLs reacted even if the APCs were fixed by 0.25% paraformaldehyde, excluding that either processing or intracellular metabolism is involved. Pulsed C1R-HLA-B*13:01 were incubated with the DDS for 1 h followed by two washing steps, no stimulation of drug-specific T-cell clones were observed for DDS. A C1R-HLA-B*13:01 stable clone was generated by means of transfection of the full-length cDNA plasmid encoding HLA-B*13:01. The endogenous peptides in HLA-B*13:01 were removed by using cold mild citric acid. After neutralizing with culture medium, the cells were incubated with β2M (4 µg/mL), GolgiStop (1 µg/mL; BD Biosciences, Calif), and the synthetic peptides for 3 h at room temperature.

### HLA-B*13:01 protein extraction, co-immunoprecipitation (Co-IP) and peptides identification by liquid chromatograph-mass spectrometer (LC–MS)

C1R and C1R-HLA-B*13:01 were cultured with DDS (50 µg/mL) for 6 h and collected cell pellet to add RIPA lysis buffer. The supernatants were collected by centrifuge at 10,000 rcf for 10 min and transferred to a 3 kD ultrafiltration tube and centrifuge at 12,000 rcf for 10 min at 4 °C, then collected less than 3 kD peptides. The protein samples were testing by HLA-ABC antibody immobilization and Co-IP assay and analyzed by Easy-nLC1000 and Q Exactive™ Hybrid Quadrupole-Orbitrap™ Mass Spectrometer (Thermo Fisher Scientific, USA). The raw MS files were analyzed and searched against protein database based on the species of the samples using PEAKS Studio. The parameters were set as follows: the protein modifications were carbamidomethylation (C) (fixed), oxidation (M) (variable), Acetyl (Protein N-term) (variable); the enzyme specificity was set to Unspecific; the maximum missed cleavages were set to 2; the precursor ion mass tolerance was set to 20 ppm, and MS/MS tolerance was 20 ppm. Only high confident identified peptides were chosen for downstream protein identification analysis.

### Growth of DDS-specific T cells

Peripheral blood mononuclear cells (PBMCs) were isolated from whole blood samples using Ficoll-Paque (GE, Life Sciences, USA) density gradient centrifugation. The PBMCs (5 × 10e5 per well) of patients with DDS-induced SCAR and other control individuals were cultured in 96-well microplates in TexMACS medium (miltenyi Biotec, Germany) supplemented with 5% human AB serum [42] (Sigma-Aldrich, Darmastadt, Germany), penicillin–streptomycin (Gibco Invitrogen, USA), 200U IL-2 (R&D systems, USA), and 50 µg/mL DDS (Sigma-Aldrich, St. Louis, MO) for 1 week at 37 °C in 5% CO_2_. DDS specific T-cell lines (TCLs) were obtained by culturing the patients’ PBMCs with DDS (50 µg/mL) for 12–14 days, and the expanded T cells were then restimulated with irradiated (50 Gy) autologous B-LCLs and DDS for approximately 4 to 5 cycles. The T-cell clones were obtained by means of serial dilution. Ten CTL TCLs were sorted by using FACSAria (BD, Franklin Lakes, NJ). In addition, dimethyl sulfoxide (DMSO) was added to the medium as the solvent control, and phytohemagglutinin (i.e., PHA) at a concentration of 10 µg/mL was used as the positive control. Proliferative clonotypes of T cells were performed to obtain for T-cell receptors (TCR) study.

### TCR repertoire analysis by high-throughput next generation sequencing and single-cell sequencing

Proliferative clonotypes of T cells from DDS-induced SCAR patients were performed for (1) normal TCR sequencing based approach, that RNA was purified from each clone using Qiagen RNeasy, and 5′RACE was performed using BD SmartRace reagents and protocol, using the universal 5′primer, and a 3′gene-specific primer for the TCR constant region. (2) For experiments using the 10 × Genomics platform, Human T Cell (PN-1000005) were used according to the manufacturer’s instructions in the Single Cell V(D)J Reagent Kits User Guide. Cell number and concentration was confirmed with TC20™ Automated Cell Counter. Approximately Number (5000, etc.) cells were subjected immediately onto the 10XGenomics Chromium Controller machine for Gel Beads-in-Emulsion (GEM) generation. Full-length V(D)J segments from either T cell transcripts are enriched from first-strand cDNA via PCR amplification with primers specific to either the TCR or Ig constant regions prior to library construction. Library quality and concentration were assessed using Agilent Bioanalyzer 2100. Libraries were run on the Hiseq X or Novaseq for Illumina PE150 sequencing. Post-processing and quality control were performed by Novogene using the 10X Cell Ranger package (v2.2.0, 10X Genomics). Reads were aligned to mm10 (or GRCh38, etc.) reference assembly (v2.2.0, 10X Genomics).

### Generation of single chain TCRα/TCRβ expression constructs and cells

Retroviruses encoding TCR genes carrying human TCR constant regions had the format TCRα-F2A-TCRβ and were produced in HEK-293T cells by transient transfection of retroviral-based plasmids and their packaging vectors (psPAX2 and pMD2.G) using lipofectamine 3000 (Thermo, Life, USA) according to the manufacturer’s protocol. At 48 h after transfection, the virus was collected, filtered through a 0.45-µm syringe filter, and used for infection. The T cells from PBMC were spin-infected with viral supernatant supplemented with 10 µg/mL Polybrene at 2500 r.p.m. at 30 °C for 90 min. On day 3 post-infection, TCR expressing cells were sorted by flow cytometry to establish derivative cell lines as indicated.

### Flow cytometry

Flow cytometry was carried out using distinct fluorochrome-conjugated mAbs that recognize human CD3, CD4, and CD8 (BD Biosciences), human TCRα, TCRβ (BioLegend). These mAbs were labeled with Alexa Fluor 488, phycoerythrin (PE), phycoerythrin–cyanine 5 (PC5), phycoerythrin-cyanin 7 (PC7) or allophycocyanine. The cells were examined by means of multicolor flow cytometry on the BD FACSVerse flow cytometer (BD Biosciences), and data were analyzed with the Flowjo™ V10 software (BD Biosciences).

### T cell activation and transduction

To transduce primary human T cells, PBMC (2 × 10e6 cells/mL) were activated in 24-well plates coated with 1 µg/mL antibody to CD3 (clone OKT3, eBioscience) in the presence of 1 µg/mL soluble antibody to CD28 (clone CD28.2, eBioscience) and 300U/mL interleukin-2 (IL-2; Fisher Scientific) in TexMACS medium supplemented with 5% (v/v) human AB serum and antibiotics (penicillin–streptomycin). After 48 h of activation, the cells were spin-infected with viral supernatant supplemented with 10 µg/mL Polybrene for 90 min at 2500 rpm at 30 °C. After spin-infection, the retroviral supernatant was replaced with fresh T cell medium containing 300 U/mL IL-2 and 1 µg/mL anti-CD28. The transduced primary T cells were cultured for 48 h and then used for cytotoxicity assays.

### ELISpot assay

PBMCs isolated from DDS-SCAR patients were incubated with DDS for 1 week. The DDS-specific T cells were then expanded by adding 50 µg/mL DDS, 30 nanogram/mL CD28, 30 nanogram/mL OKT3 (anti-CD3 antibody), and 200 U/mL IL-2 for 2 weeks. The expanded T cells were reset for 3 days, and CTLs were isolated using FACS with anti-human CD45, CD3, and CD8 antibodies as markers. The DDS-specific CTLs were detected by quantifying the IFN-γ release with ELISpot assay following the instructions of the human IFN-γ ELISpot kit (BD Biosciences, USA). To perform ELISpot analysis, 5** × **10e4 DDS-specific CTLs or TCR transfectants were incubated with 1** × **10e4 C1R, C1R-HLA-B*13:01 or EBV-B-LCL-13:01 APC at 37 °C for 24 h. Data was analyzed using the CTL ELISpot Reader (CTL, USA).

### Real-time RT-PCR assay

Total RNA extracted from patients’ PBMCs using Qiagen RNA Extraction Kit and was reverse transcribed using the Superscript II reverse transcriptase enzyme (Life Technologies). RT-PCR was performed in 20 µL reactions containing 0.8 µM forward and reverse primers and 5 µL of extracted RNA on an ABI 7300 (Thermo Fisher) using the AgPath-ID One-Step RT-PCR kit (Thermo Fisher). Thermocycle conditions included initial denaturation at 50 °C and 95 °C (10 min each), followed by 40 cycles at 95 °C (15 s) and 60 °C (1 min). The TCR primers used in quantitative-PCR amplification were showed in Additional file 3: Table S1. To verify primer specificities, melting curve analyses and PCR product sequencing were performed. Ct value were calculated via 2-ΔΔct method after normalization to GAPDH.

### Modeling TCR/peptide/HLA and DDS

The 3D structures of HLA-B*13:01 and a TCR clonotype were first constructed with homology modeling method Modeller independently [43]. And next the complex structure was assembled by referring the existing crystal structure (PDB code: 4EUP) using structure alignment program TMalign [44]. This complex was further refined by a protein side-chain repacking method CISRR [45] as well as energy minimization based on CHARMM22 force field [46]. The binding modes of DDS with the HLA-B*1301 were generated by protein–ligand blind docking tool CB-Dock [47] and superposed onto the complex structure of HLA-B*13:01-TCR. Images were generated with PyMOL (PyMOL Molecular Graphics System, Version 1.2, Schrodinger LLC, New York, NY).

### Determination of inflammatory cytokine and cytotoxin production levels

To quantify IFN-γ, IL-3, IL-13, IL-5, IL-21, granulysin and granzyme B production levels, B-LCL (HLA-B*13:01) were plated in 24-well plates (10e5 cells/well) in co-culture with CTL (5 × 10e5 cells/well) and stimulated with DDS (50 µg/mL) in 1 mL complete RPMI1640 media overnight. Afterward, the supernatant concentrations of the inflammatory cytokines and cytotoxins mentioned above were quantified with ELISA (R&D, USA).

### Statistical analysis

Statistical analyses were performed using SPSS for Windows, version 21.0 (IBM, Armonk, NY) and GraphPad prism 8.0 (San Diego, CA). Heatmaps were created using the built-in R heatmap function in stats package, and the Circos plots were generated using the VDJtools software (MiLaboratory). The entire experiment was repeated thrice. P-values for ratio estimates were calculated using a two-sided test. For analysis of ELISpot results, statistical comparison between two variables was performed by two-tailed Student’s t-test. Differences were considered statistically significant at P-values of less than 0.05.

### Data availability

All sequencing data obtained in the study have been deposited in the NCBI sequence read archive (SRA) database with links to BioProject accession ID “PRJNA867422” (it will be uploaded after manuscript accepted). The atomic coordinates and structure factors of the HLA-B*13:01-β2M-peptide complex was deposited in the Protein Data Bank under the accession number 7ER5. All other data are available from the authors upon reasonable request.

## Results

### Study participants

Twelve HLA-B*13:01-positive DDS SCAR patients were enrolled in this study. The underlying conditions caused by treatment with DDS were mostly diagnosed as chronic inflammatory dermatoses, including exfoliative dermatitis, erythroderma and eosinophilia (Table 1). The average daily DDS dose was 90.0 ± 20.4 mg, and the average onset of cutaneous manifestations was 29.2 ± 15.7 days after exposure to DDS. In addition, ten DDS-tolerant individuals (three HLA-B*13:01-positive and seven HLA-B*13:01-negative) who had been administered DDS for at least 3 months without any cutaneous adverse drug reaction, and twelve healthy controls were also recruited. All study participants were of Chinese descent.

### DDS-specific cytotoxic T lymphocytes (CTLs) are activated by DDS in HLA-B*13:01-dependent manner

**Fig. 1:**
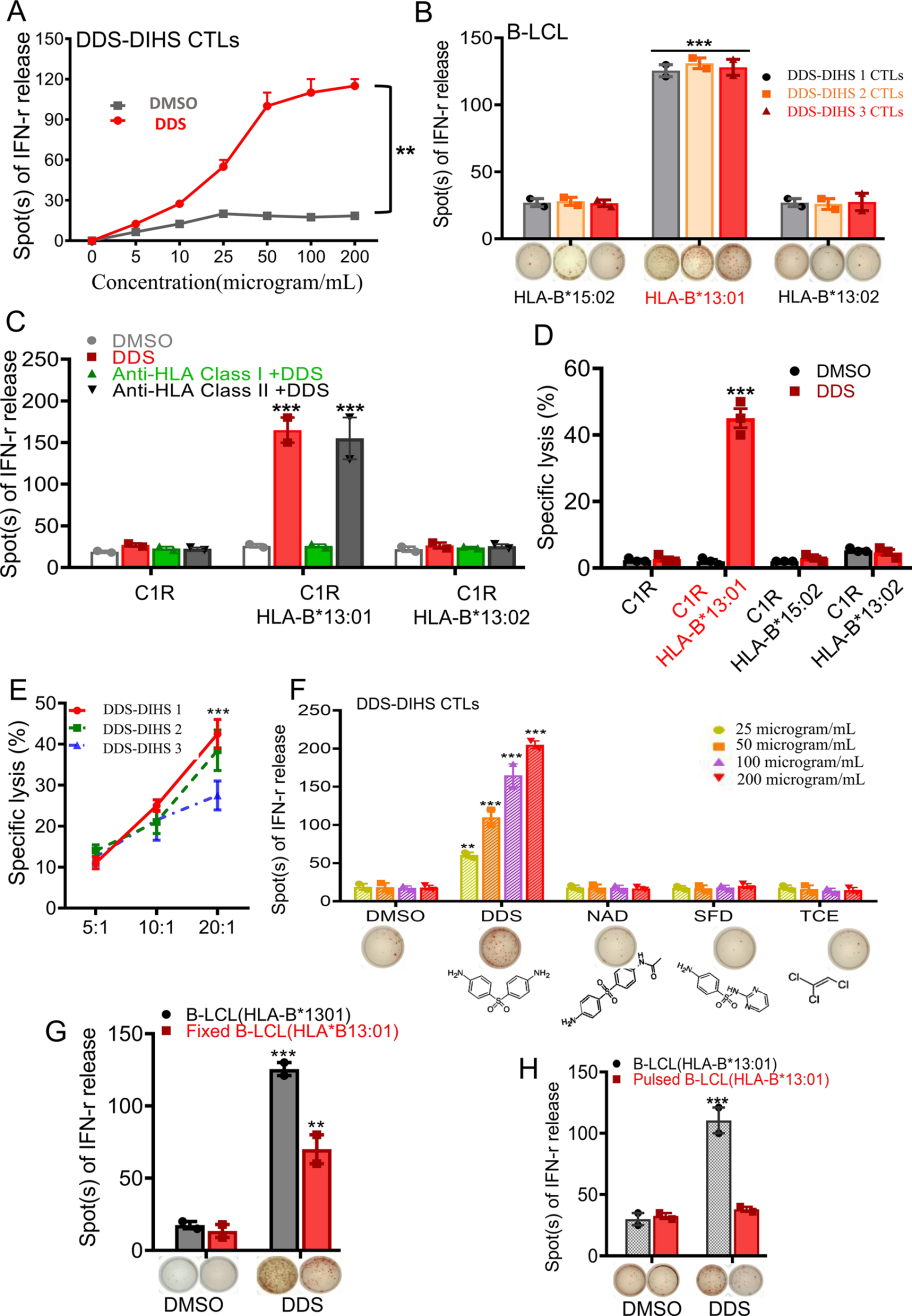
HLA-B*13:01-dependent activation of DDS-specific CTLs in patients with DDS-induced DIHS. **A** T lymphocyte activation test was performed by IFN-γ ELISpot release assay for CTLs expanded from PBMC of 5 DDS-induced DIHS patients cultured with DDS or DMSO (solvent control). The CTLs-mediated IFN-γ release displayed in a DDS concentration manner. **B** allogeneic HLA-B*15:02 and HLA-B*13:02 carriers, then in vitro cultured with CTLs from three patients with DDS-induced DIHS upon DDS treatment. **C** HLA blocking assay were performed, and the IFN-γ releases of DDS-specific CTLs against different cell lines (C1R, HLA-B*13:01 expressing B-LCL or HLA-B*13:02 expressing B-LCL) in the absence or presence of HLA class I or class II antibodies are shown. **D** DDS-specific CTL induced HLA-B*13:01 expressing B-LCL cell death, but not induced C1R, HLA-B*13:02 expressing B-LCL or HLA-B*15:02 expressing B-LCL, as measured by means of annexin-V labeling for flow cytometry assay. **E** Cytotoxicity of DDS-specific CTLs against HLA-B*13:01 expressing B-LCL in the presence of DDS as the concentration increases. **F** DDS-specific CTLs against HLA-B*13:01 expressing B-LCL transfectants and DDS structure/ HLA-B*13:01-related chemicals, including N-Acetyl DDS (NAD), Sulfadiazine (SFD), and Trichloroethylene(TCE). Values are mean and standard error of the mean. **G** T lymphocyte activation test was performed the IFN-γ release from PBMC -fixing assays cultured with DDS or DMSO. **H** Activation of CTL cells were analyzed by C1R (HLA-B*13:01)-washing assays cultured with DDS or DMSO respectively. The entire experiment was repeated thrice. *P < 0.05, **P < 0.01, by two-tailed Student t test. B-LCL, B-lymphoblastoid cell line, CTL, cytotoxic T lymphocyte; DIHS, dapsone-induced hypersensitivity syndrome; PBMC, peripheral blood mononuclear cell

We isolated PBMCs from all DDS-DIHS patients and tolerant individuals, performed in vitro T cell expansion in the presence of 50 µg/mL DDS. After approximately three to four cycles of co-culture, CD8^+^CTLs constituted the majority of the T cell population in samples originating from the DDS-DIHS patients, but were largely absent in the T cell populations of tolerant individuals (Additional file 2: Fig. S1A–C). Thus, DDS-specific CD8^+^CTL clones could be obtained from DDS-DIHS patients and maintained in vitro*.* We then verified the dependence of DDS-specific CTL activation on the presence of different HLA-B alleles and stimulation by DDS or other drugs. The activation was detected using IFN-γ ELISPOT assay in the manner dependent on DDS concentration (Fig. 1A, Additional file 2: Fig. S2A). Under the conditions of DDS stimulation, DDS-specific CTLs were activated when co-cultured with autologous HLA-B*13:01-expressing B-lymphoblastoid cell line (B-LCL), but not with allogeneic HLA-B*15:02- or HLA-B*13:02- expressing B-LCL (Fig. 1B). To further confirm HLA-B*13:01-specific activation of CTLs in DDS-DIHS, we performed HLA blocking assay using DDS-specific T-cells obtained at the end of the second cycle (14 days) of clonal expansion. The results indicated that CTL-mediated IFN-γ release could be blocked by anti-HLA class I antibodies but not by anti–HLA class II antibodies (Fig. 1C, Additional file 2: Fig. S2B). Moreover, the cytotoxicity effect of DDS-specific CTLs by flow cytometric with PI/annexin V was dependent on both DDS and HLA-B*13:01 (Fig. 1D), and was significantly intensified with the increase of the effector/target cell ratios at 20:1 (Fig. 1E). Furthermore, the IFN-γ release from CTLs of DDS-DIHS patients was observed in a DDS concentration-dependent manner, but not in sulfadiazine (SFD), the DDS metabolite N-acetyl DDS (NAD), trichloroethylene (TCE) or in solvent control (DMSO) (Fig. 1F). Further investigated the cross-reactivity of dapsone and other sulfa drugs in 6 dapsone-induced SCAR patients carried HLA-B*13:01, the result showed that dapsone-induced SCAR patients carried HLA-B*13:01 had a high cross-reactivity to sulfasalazine and sulfamethoxazole (sensitivity 66.7% and 83.3%, respectively), but not to probenecid, gliclazide and glimepiride (Additional file 2: Fig. S3A and B). This result was consistent with the clinal and genetic finding. We then evaluated antigen presentation in DDS-DIHS patients using APC-fixing assays. DDS-elicited activation of CTLs could not be completely abolished by fixing APCs with 0.25% paraformaldehyde (Fig. 1G), but was abolished by a pulsed procedure (Fig. 1H). Altogether, these results demonstrated that expanded CTLs derived from DDS-DIHS patients are activated by DDS in HLA-B*13:01-dependent manner.

### HLA-B*13:01 binds DDS directly

To determine how HLA-B*13:01 and DDS interact, we first expressed recombinant HLA-B*13:01 protein in *Escherichia coli* and purified it for surface plasmon resonance experiments, and then with the β2-microglobulin form the complex (Additional file 2: Fig. S4A). β2-microglobulin could bound to HLA-B*13:01 protein based on IP assay (Additional file 2: Fig. S4B). The results showed that the recombinant HLA-B*13:01 directly binds DDS with an estimated binding affinity in the micromolar range (Kd = 68.54 µM) (Additional file 2: Fig. S4C). To further investigate whether the endogenous peptides were involved in the HLA-B*13:01-mediated presentation of DDS, HLA-B*13:01 was expressed in the C1R B-cells (HLA-B-deficient cells) by stable transfection. Afterwards, the lysates were collected from HLA-B*13:01-expressing C1R cells and wild-type C1R cells to identify HLA-B*13:01-binding peptides with liquid chromatography-mass spectrometry (LC–MS). Considering that HLA-B*13:01 belongs to HLA class I receptors that preferentially bind 9–12-mer peptides, we used ultrafiltration tubes with the cut-off molecular weight of 3 kDa to collect the endogenous peptides. A total of seven peptides were detected in samples from HLA-B*13:01-expressing C1R cells significantly high expression than wild-type C1R cells. Among these peptides, VGFIGAGQLAF and VGFIGAGQLAY were expressed highly in HLA-B*13:01-expressing C1R cells in the presence of DDS (Additional file 2: Fig. S5A). These two abundant endogenous peptides elicited IFN-γ release from HLA-B*13:01-expressing cells in the presence of DDS, but not wild-type C1R cells (Additional file 2: Fig. S5B). However, treatment with excessive amounts of either of these two peptides did not strengthen the T cell activation when compared to DDS stimulation alone (Additional file 2: Fig. S5B). These results reveal that HLA-B*13:01 directly binds DDS, and that the presentation of endogenous peptides is suggested to be dispensable for binding of DDS to HLA-B*13:01 itself.

### Key residues in the DDS-binding groove of HLA-B*13:01

**Fig. 2:**
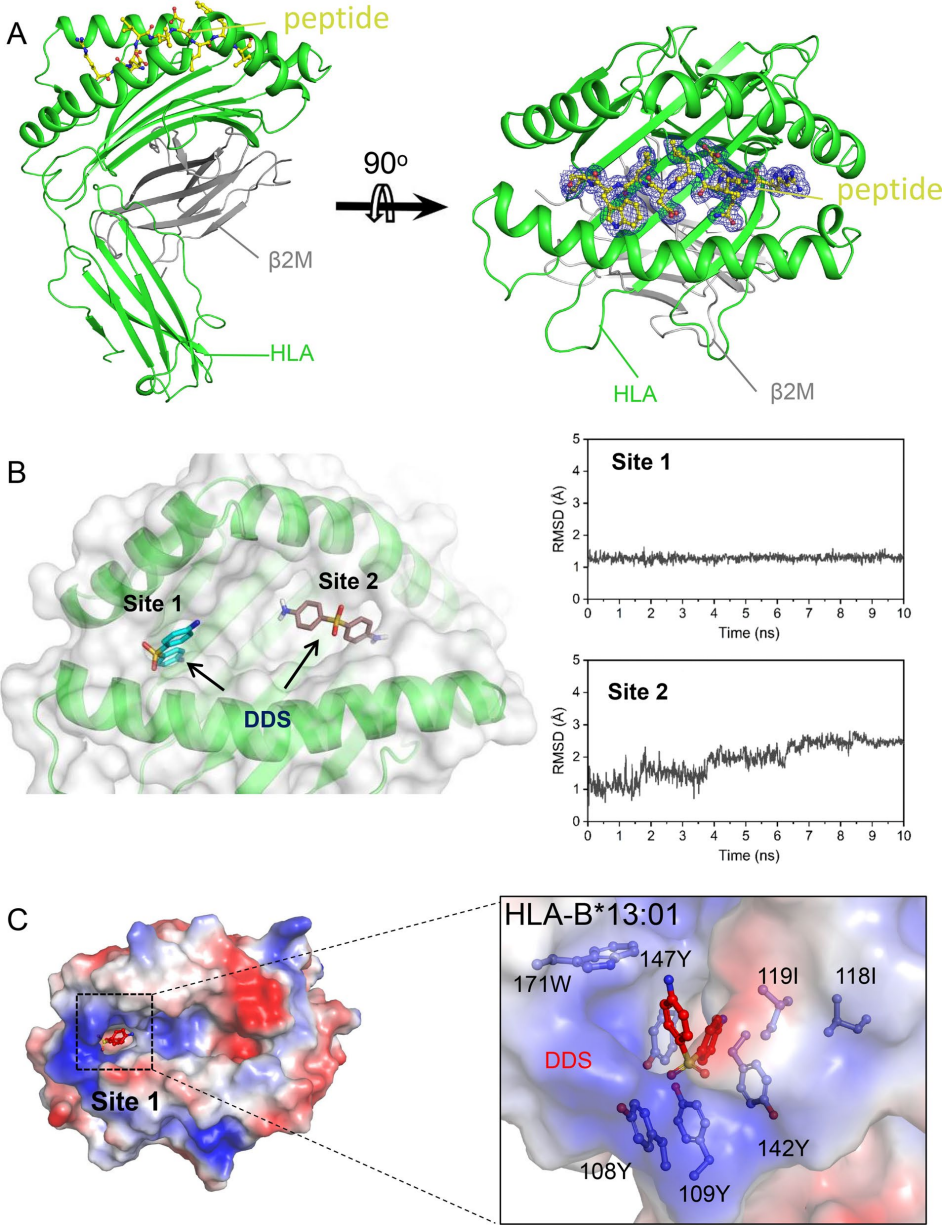
HLA-B*13:01 protein directly presents DDS without intracellular metabolism and the overall structure of HLA-B*13:01-β2M-peptide. **A** The crystal structure of HLA-B*13:01**-**β2M**-**peptide was determined at resolution of 1.50 Å. Ribbon representation of HLA-B*13:01**-**β2M and stick representation of the peptide. In HLA-B*13:01, HLA is shown in green, β2M is shown in gray and the peptide is shown in yellow. The 2Fo-Fc map of the peptide contoured at 1.2 σ. **B** DDS potentially binds to the F pocket of binding groove due to a constant RMSDs value. **C** The pocket where the DDS is located in HLA-B*13:01. Electrostatic surface representation of the pocket of DDS and the residues that make up this pocket are shown as stick in HLA-B*13:01

To gain deeper insight into the mechanisms by which HLA-B*13:01 binds DDS, we had tried to crystallize a complex of HLA-B*13:01-β2M with peptide and DDS, but most of the proteins precipitated after adding DDS and no crystals of the complex was obtained. But we determined the crystal structure of HLA-B*13:01-beta-2-microglobulin (β2M)-peptide complex at 1.50 Å resolution (Additional file 1: PDB ID 7YG3). The peptide which predicted by the NetMHC 4.0 server, was used to stabilize the structure of HLA-B*13:01-β2M and facilitate crystallization but not participate in DDS interaction. HLA molecules are heterodimers composed of α and β chains, and the main differences lie in the composition of peptide binding region. The peptide binding region of HLA class I consists of α1 and α2 domains of α chain, while this region of HLA class II consists of α1 domain of α chain and β1 domain of β chain. The HLA-B*13:01-β2M-peptide complex is typical of HLA class I complex (Fig. 2A). We further showed that DDS potentially binds to the F pocket of binding groove (termed site 1), which appeared to be stable due to a constant root-mean-square deviation (RMSDs) value (Fig. 2B). For in-depth analysis of binding of DDS to the site 1, we conducted molecular modeling using Accelrys Discovery Studio. The results revealed that DDS is buried in a hydrophobic pocket formed by 108Y, 109Y, 118I, 119I, 142Y, 147Y and 171W of HLA-B*13:01 (Fig. 2C).

**Fig. 3:**
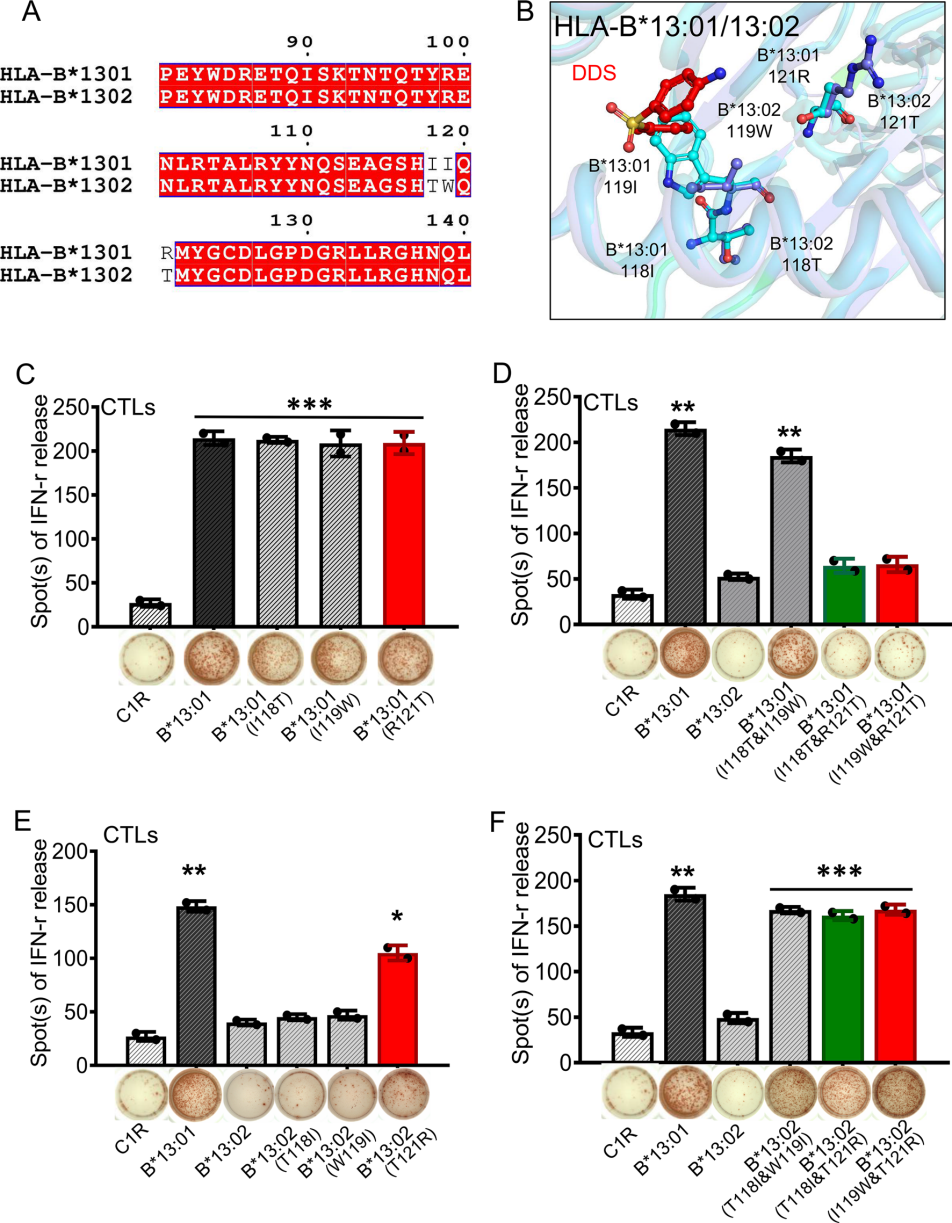
Key residues of HLA-B*13 family members are involved in DDS presentation and CTLs-mediated activation. **A** the difference between HLA-B*13:01 and HLA-B*13:02 in amino acid sequence. **B** the difference between HLA-B*13:01 and HLA-B*13:02 in structure. 121R of HLA-B*13:01 and 121T of HLA-B*13:02 with DDS complex are shown as stick and electrostatic surface representation, respectively. **C** the IFN-γ releases of CTLs expanded from DDS-induced DIHS patients against DDS and C1R B transfectants expressing different HLA-B*13:01 wild type or single-site mutants. **D** the IFN-γ releases of CTLs against DDS and C1R B transfectants expressing different HLA-B*13:01 wild type, HLA-B*13:02 wild type, or two single-site mutants of HLA-B*13:01. **E** the IFN-γ releases of CTL against DDS and C1R B transfectants expressing different HLA-B*13:01 wild type, HLA-B*13:02 wild type, or single-site mutants of HLA-B*13:02. **F** the IFN-γ releases of CTL against DDS and C1R B transfectants expressing different HLA-B*13:01 wild type, HLA-B*13:02 wild type, or two single-site mutants of HLA-B*13:02. The entire experiment was repeated thrice and values are mean standard error of the mean. *P < 0.01; **P < 0.05; ***P < 0.001, by two-tailed Student *t* test

HLA-B*13:01 shares 98% of sequence identity with HLA-B*13:02, and only three amino acid residues differ (118I_B13:01_/118T_B13:02_, 119I_B13:01_/119W_B13:02_, 121R_B13:01_/121T_B13:02_) (Fig. 3A). Nevertheless, DDS-DIHS is strongly associated with HLA-B*13:01 but not HLA-B*13:02, implying that the identity of these three residues plays an important role in DDS binding. Indeed, we found that the side chain of 119W_B13:02_ creates steric hindrance with DDS (Fig. 3B). Besides, the van der Waals interactions that 118T_B13:02_ forms with adjacent amino acids, including 119W_B13:02_, are stronger than those formed by 118I_B13:01_. Thus, 118T_B13:02_ and 119W_B13:02_ work together to obstruct DDS binding by changing the shape of the binding pocket (Figs. 2C, 3B). While there is no significant difference between conformations of 121R_B13:01_ and 121T_B13:02_, these two residues differently affect the electrostatic properties of the DDS-binding pocket, indicating that 121R_B13:01_ may also play an important role in DDS binding (Fig. 3B).

To verify the importance of the aforementioned residues for DDS binding, we generated C1R cell lines stably expressing single-site and double-site HLA-B*13:01 mutants (HLA-B*13:01^I118T^, HLA-B*13:01^I119W^, HLA-B*13:01^R121T^, HLA-B*13:01^I118T&I119W^, HLA-B*13:01^I118T&R121T^, and HLA-B*13:01^I119W&R121T^) and HLA-B*13:02 mutants (HLA-B*13:02^T118I^, HLA-B*13:02^W119I^, HLA-B*13:02^T121R^, HLA-B*13:02^T118I&W119I^, HLA-B*13:02^T118I&T121R^, and HLA-B*13:02 ^I119W&T121R^), and used them to perform in vitro co-culture assays with DDS-specific CTLs expanded for two weeks. The CTL-mediated IFN-γ release was measured for each mutant variant by ELISpot assay (Fig. 3C–F). As expected, the levels of IFN-γ released from DDS-specific CTLs were significantly elevated upon co-culture with HLA-B*13:01-expressing APCs and stimulation with DDS (ninefold increase compared with co-culture with wild-type C1R cells, P < 0.01, Fig. 3C). Interestingly, CTL activation was not significantly altered when co-cultured with HLA-B*13:01^I118T^, HLA-B*13:01^I119W^, or HLA-B*13:01^R121T^ (Fig. 3C), but was greatly decreased in co-cultures with the double-site mutants HLA-B*13:01^I118T&R121T^ and HLA-B*13:01^I119W&R121T^ (Fig. 3D). Correspondingly, the single-site mutants HLA-B*13:02^T118I^ and HLA-B*13:02^W119I^ did not noticeably change CTL activation (Fig. 3E), whereas the single-site mutant HLA-B*13:02^T121R^ and the two-site mutants HLA-B*13:02^T118I&W119I^, HLA-B*13:02^T118I&T121R^, and HLA-B*13:02^I119W&T121R^ significantly increased CTL activation (Fig. 3E, F). Altogether, these results demonstrate that I118 or I119 and R121 are key residues for DDS binding by HLA-B*13:01.

### TCR sequencing confirms poly- and mono-clonality of DDS-specific T cells

**Fig. 4:**
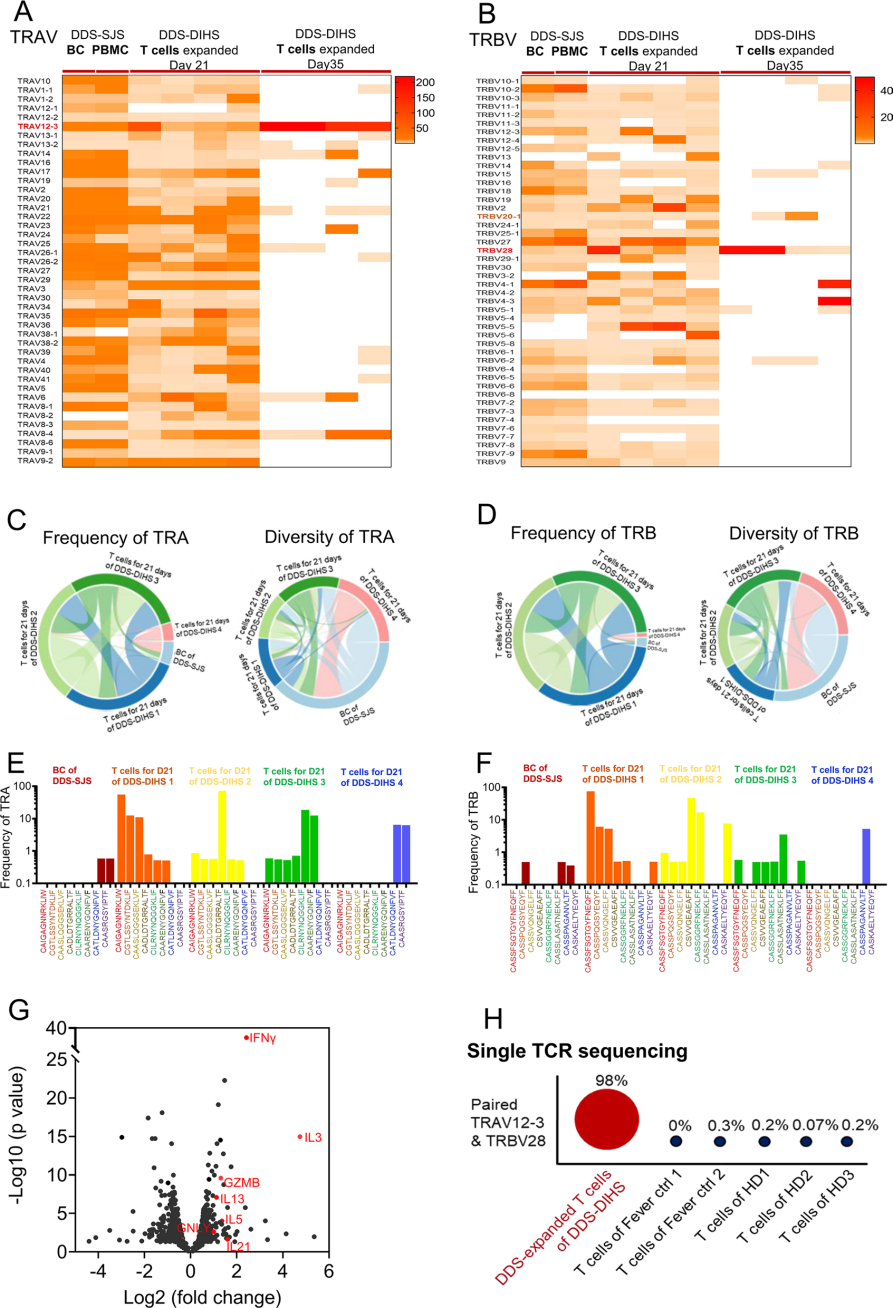
Identification of DDS-specific TCR in patients with DDS-induced SCAR. **A, B** analysis of 5′RACE-based TCR sequence results were performed in parallel for UMI corrected data. The expression profiles of TCR repertoire in the blister cell and active PBMC from one DDS-induced SJS patients as well as T cells expanded for 21 days or 35 days of 4 DDS-DIHS patients were analyzed by next-generation sequencing. Heatmaps display the expression values of the TRAV and TRBV genes from each sample, which have been normalized to the mean values of the corresponding gene of the 12 healthy donors’ PBMC, respectively. The definition of TRBV and TRBJ was based on the IMGT database. TRAV12-3, TRBV28, and TRBV20-1 were the most frequent TCR V genes in T cells from patients with DDS-induced SCAR. **C, D** the pairwise overlap Circos plot shows the overlapping frequency and diversity of TRA and TRB clonotypes among the samples of DDS-induced SCAR, respectively. **E, F** the frequency of TRA and TRB CDR3 sequence among the samples of DDS-induced SCAR, respectively. The different bar of color represents the frequency of TCR CDR3 in different DDS-induced SCAR patients. **G** A volcano plot of DEGs that are upregulated or downregulated in DDS-expanded T cells from 5 DDS-induced SCAR patients comparing to non-expansion T cells of 5 healthy donors. The DDS-expanded T cells or non-expansion T cells were collected by FACS using CD3 antibody. The red points and gene names represent the SCAR-related genes as reported previously. P value of volcano plot was derived by Wilcoxon rank-sum test. **H** single-cell TCR sequencing were performed by 10XGenomics in samples, including DDS-expanded T cells from 1 DDS-induced DIHS patient, non- expanded T cells from 2 fever patients and non- expanded T cells form 3 healthy donors. The frequency of paired TRAV12-3/TRBV28 were shown. The larger size of the circle represents the higher frequency of TCR. Ctrl, control; DEGs, differentially expressed genes; DIHS, drug-induced hypersensitivity syndrome; FACS, Fluorescence activated cell sorter; HD, healthy donors; SJS, Stevens-Johnson syndrome; SCAR, Severe cutaneous adverse reaction; TCR, T cell receptor

TCRs are essential for recognition of HLA-drug complex and T cell activation. To analyze 5′ RACE-based TCR sequencing results in parallel with UMI-corrected data, we firstly characterized the distribution of TCRα variable (TRAV) and TCRβ variable (TRBV) genes across blister cells and active T cells of DDS-induced SJS, as well as T cells derived from DDS-DIHS patients (Fig. 4A, B), and CD8^+^ T cell populations were significantly overexpressed over time for 21 or 35 days (Additional file 2: Fig. S1A–C). The most abundant transcripts included TRAV12-3, TRBV20-1, and TRBV28, which were all strongly correlated with increases in the blister cells, active PBMCs, as well as DDS-expanded T cells from DDS-SCAR patients, but not with the PBMCs from twelve healthy controls (Fig. 4A, B). We then mapped the circos plot for TCR CDR3 assemblies, and found a high level of homology and shared sequence identity within TRA/TRB CDR3 regions of DDS-DIHS patients (Fig. 4C, D). The shared specific TCR CDR3 clonotypes were oligoclonally expanded after 21 days of DDS stimulation, but were absent or present at very low frequencies (< 0.001%) in PBMCs of twelve healthy controls (Fig. 4E, F). Although the clonotype frequency varied, DDS-DIHS-1, DDS-DIHS-2, and DDS-DIHS-3 patients shared the TRA CDR3 clonotypes ‘CAIGAGNNRKLIW’, ‘CGTLSSYNTDKLIF’, ‘CAASLQGGSEKLVF’, ‘CADLDTGRRALTF’, ‘CILRNYNQGG KLIF’, ‘CAARENYGQNFVF’ (Fig. 4E), and the TRB CDR3 clonotypes ‘CASSFSGTGYFNEQFF’, ‘CASSPQGSYEQYF’, ‘CASSVQNGELFF’, ‘CSVVGEAEAFF’, ‘CASSGGRFNEKLFF’ (Fig. 4F). In addition, the TRA CDR3 clonotypes ‘CATLDNYGQNFVF’ and ‘CAASRGSYIPTF’ (Fig. 4E), and the TRB CDR3 clonotype ‘CASSPAGANVLTF’ (Fig. 4F) were expanded in DDS-DIHS-4 patient and were also detected in blister cells of DDS-induced SJS.

To investigate the gene expression profile associated with the aforementioned TCR clonotypes, we performed RNA-Seq and analyzed the differentially expressed genes (DEGs) of DDS-expanded T cells from five DDS-DIHS patients in comparison to unexpanded T cells of five healthy controls (Fig. 4G). The expression levels of IFN-γ, IL-3, IL-13, IL-5, IL-21, granulysin and granzyme B, all of which have been associated with pathogenesis of SJS and DDS-DIHS [48–52], were elevated in the DDS-expanded T cells expressing the aforementioned TCR clonotypes (Fig. 4G), further suggesting that these TCR clonotypes are involved in the pathogenesis of DDS-DIHS.

We next performed single-cell TCR sequencing to determine TRAV-TRBV pairing in T cells isolated from one DDS-DIHS patient (Fig. 4H). The results indicated that 98% of DDS-expanded T cells expressed the paired TRAV12-3/TRBV28 with the TRA CDR3 “CAASRGSYIPTF” clonotype (Fig. 4H). In contrast, only up to 0.3% of T cells from five healthy controls expressed the paired TRAV12-3/TRBV28, and none expressed the TRA CDR3 “CAASRGSYIPTF” clonotype. This finding indicated that T cells expressing the TCR composed of paired TRAV12-3/TRBV28 with TRA CDR3 ‘CAASRGSYIPTF’ clonotype are responsible for T cell activation and hypersensitivity reactions in DDS-DIHS.

### Determination of the DDS-specific immunodominant TCR V clonotype and its role in pathogenesis of DDS-DIHS

**Fig. 5:**
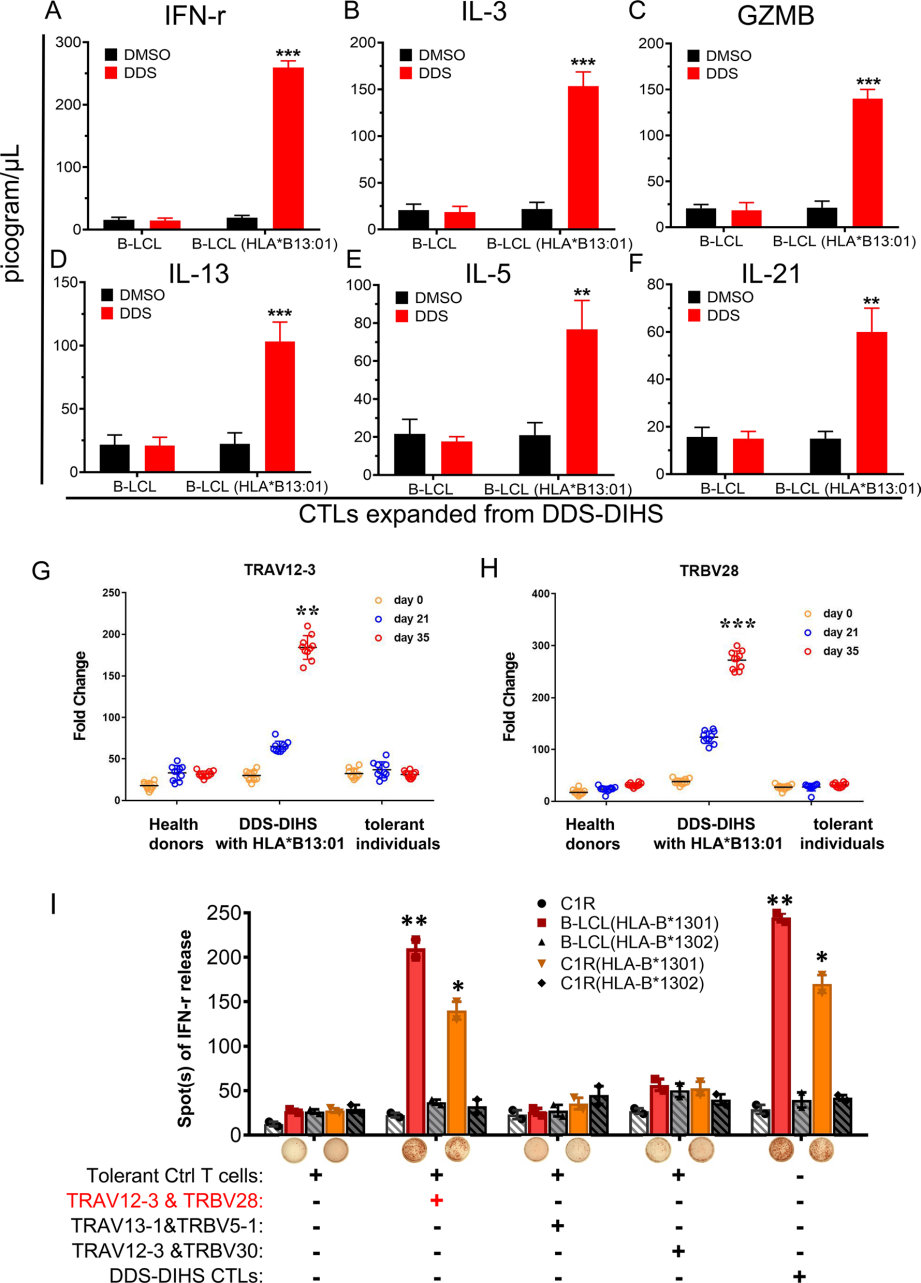
Immune response of the DDS-specific TCR transfectants to DDS and HLA-B*13:01. **A–F** ELISA was used to quantify the amount of IFN-γ, IL-3, IL-13, IL-5, IL-21 and granzyme B in DDS and DMSO stimulated CTL. **G, H** the expression levels of TRAV12-3 and TRBV28 were determined by qPCR assay, respectively. 10 patients with DDS DIHS, 10 dapsone tolerant control, and 10 health donors were enrolled. Fold change were calculated via 2^−ΔΔct^ method after normalization to GAPDH. **I** IFN-γ ELISPOT response was observed in the co-cultures of PBMC with TCR transfectants, C1R-B*13:01 or EVB-HLA-B*13:01 cells. These results suggested that the special αβTCR reacts to DDS and its structural analogs, and the HLA-B*13:01 presenting promotes the immune recognition. The entire experiment was repeated thrice. *P < 0.05, **P < 0.01, ***P < 0.001, by two-tailed Student t test

We next determined the levels of inflammatory cytokines in DDS- and B-LCL (HLA-B*13:01)-stimulated CTLs. The results showed that IFN-γ, IL-3, IL-13, IL-5, IL-21 and granzyme B levels in the DDS- and B-LCL (HLA-B*13:01)-stimulated CTLs were significantly higher than in the mock group (Fig. 5A–F). We then performed qPCR assay to examine the expression levels of TRAV and TRBV clonotypes of T cells isolated from DDS-DIHS patients. Compared to ten healthy controls and ten DDS-tolerant controls, TRAV12-3 and TRBV28 expression levels in T cells from ten DDS-DIHS patients were approximately twofold and ninefold higher on the days 21 and 35 of in vitro expansion under DDS stimulation, respectively (Fig. 5G, H). Moreover, TRAV12-3 and TRBV28 clonotypes were found in HLA-B*13:01- positive DDS-DIHS patients, but were absent in all three DDS-tolerant HLA-B*13:01-positive control subjects. Thus, the absence of the TRAV12-3 and TRBV28 clonotypes in DDS-tolerant control subjects might explain why DDS-DIHS does not always develop in all HLA-B*13:01-positive persons taking DDS.

To further verify the role of the TRAV12-3 and TRBV28 clonotypes in pathogenesis of DDS-DIHS, we overexpressed the paired TRAV12-3/TRBV28, TRAV13-1/TRBV5-1 and TRAV12-3/TRBV30 in CD3 + T cells sorted after 48 h of expansion of PBMCs isolated from DDS-tolerant controls lacking these TCR clonotypes (Additional file 2: Fig. S6). Afterward, we performed in vitro co-culture assay and found that only the co-cultures of the TRAV12-3/TRBV28 T cells with C1R-HLA-B*13:01 or B-LCL-HLA-B*13:01 produced IFN-γ upon DDS stimulation (Fig. 5I). Together, these results further confirm that the TCR with paired TRAV12-3/TRBV28 clonotype is required for formation of immune synapse with HLA-B*13:01-DDS complex and DDS-DIHS-associated T cell activation.

### The modeling of the HLA-B*13:01-DDS-TCR immune synapse

**Fig. 6:**
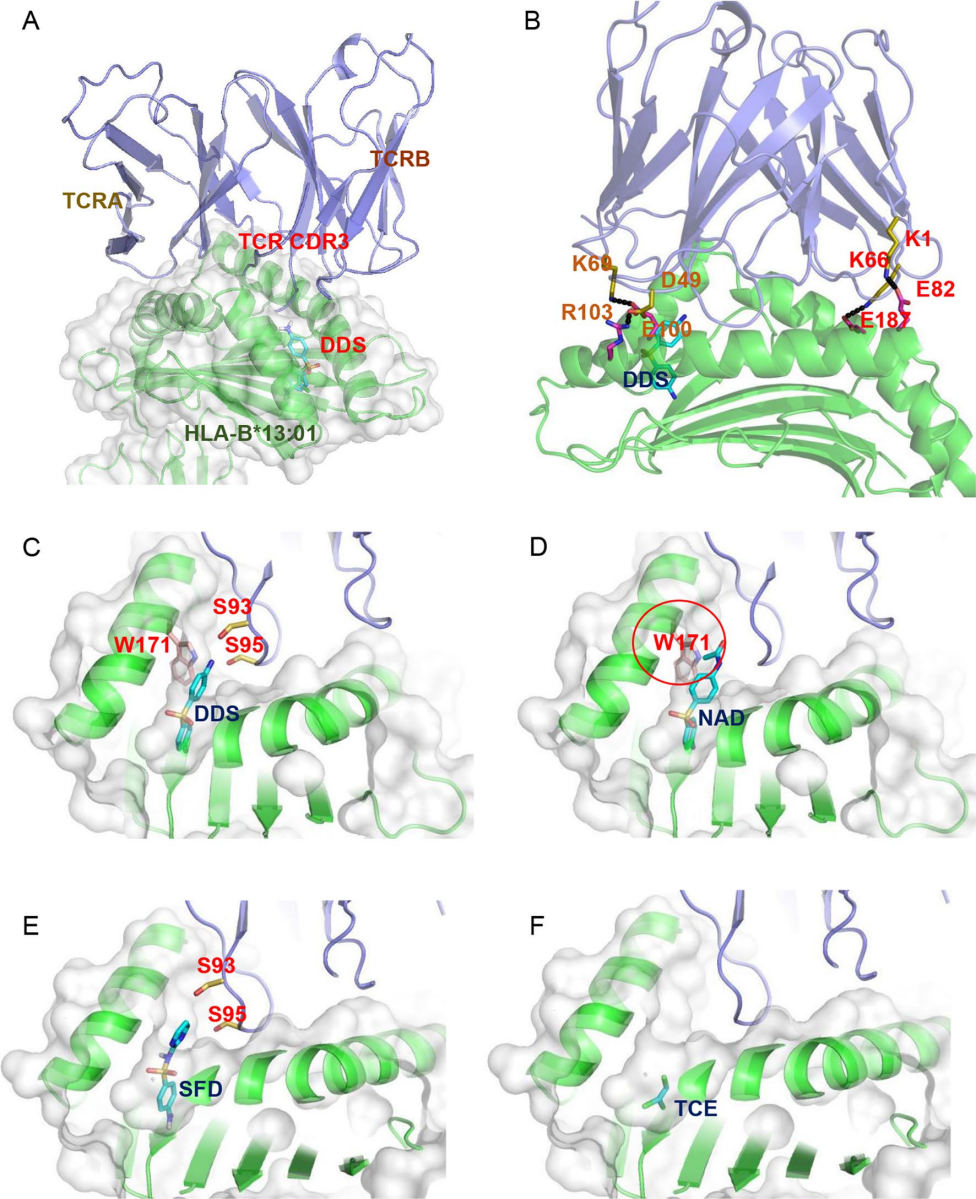
The 3D structure of HLA-B*13:01 binding with DDS and TCR. **A** TCR was binding with HLA-B*13:01 and DDS as an intermedia was drawn in stick model while HLA-B*13:01 was shown in green cartoon. **B** TCR showed multiple salt-bridges with HLA-B*13:01. The alpha and beta chain of TCR were shown in purple. **C–F** the molecular of DDS, NAD, SFD and TCE were docked with HLA-B*13:01 and TCR respectively. Both SFD, TCE and NAD cannot bind in the F pocket. All drugs were drawn in stick model while HLA-B*13:01 and TCR was shown in green and purple cartoon model, respectively

According to the results described above, DDS interacts with HLA-B*13:01 directly and is required for the formation of immune synapse with the DDS-DIHS-associated TCR clonotype. However, how does DDS bind to HLA and mediate the formation of the immune synapse with TCR without a peptide remain unclear. To answer the first question, we carried out independent in silico structure modeling of HLA-B*13:01 and TCR, followed by blind docking of DDS and HLA-B*13:01 and assembly based on the existing crystal structure (PDB ID: 4EUP) (Fig. 6A). The obtained HLA-B*13:01-DDS-TCR complex shows that the terminal amino-group of DDS contacts the residue S95 from the H-CDR3 region of TCR, possibly forming an intermolecular hydrogen bond. Concurrently, TCR forms multiple salt bridges with HLA-B*13:01 (Fig. 6B). This implies that both HLA-B*13:01 and DDS contribute to interaction with TCR and that, despite being significantly smaller than a 9-mer peptide, DDS mimics antigenic peptide due to dual interaction with TCR and HLA-B*13:01. We then performed blind docking of NAD, SFD and TCE with HLA-B*13:01, respectively. Among the three molecules, SFD and TCE showed favorable binding modes similar to DDS (Fig. 6E, F), whereas NAD was unable to bind to the F pocket of HLA-B*13:01 (Fig. 6D). Superimposition of the 3D structure of NAD over DDS in the F pocket indicated that Trp171 of HLA-B*13:01 clashes with the N-acetyl moiety of NAD (Fig. 6D), explaining the lack of interaction. Conversely, SFD showed almost the same binding mode as DDS but exposed its hydrophobic aromatic terminal to the CDR3 of TCR (Fig. 6E). This would make the interaction between SFD and TCR energetically unfavorable because the related CDR3 region consists of multiple hydrophilic residues, such as serines. Furthermore, TCE was also too small to form interactions with TCR (Fig. 6F).

## Discussion

Drugs can potentially be recognized as foreign antigens and trigger cell-mediated adaptive immune responses such as SCARs [3, 12]. Although HLA is known as an important immune receptor involved in drug presentation, few studies have investigated the structural basis for interactions between HLA and SCAR-associated drugs. Recently reported the promiscuous immune response induced by HLA Class-II‒restricted T cells in DDS-DIHS patients [21], but the detailed interaction and mechanism of HLA-B*13:01/DDS-restricted CTL responses remain not fully understood. Furthermore, the previously study used homology modeling to explore the interaction between HLA-B*13:01 and DDS [53]. Our finding demonstrate that DDS binds directly to both HLA-B*13:01 and the DDS-specific TCR, and validate key HLA-B*13:01 residues mediating DDS binding. Thus, our findings show that the HLA-B*13:01-DDS-TCR interaction preferred to the novel p-i HLA/TCR model, in which drug directly interact with HLA and TCR, and peptide does not directly participate in the HLA-drug interaction, as the classic p-i model only focused on HLA and drug interaction in antigen-processing pathway [54, 55]. It suggested that TCR also plays an important role and can directly participate in HLA-drug interaction without any peptide involvement.

According to data presented in this study, DDS is almost entirely buried within a hydrophobic pocket located in the peptide-binding groove of HLA-B*13:01. Such binding mode differs from the way by which abacavir binds to HLA-B*57:01 [56], but is comparable to the interaction between allopurinol/oxypurinol and HLA-B*58:01 [8, 57]. Furthermore, the structure of DDS appears to be critical for its interaction with HLA-B*13:01 and TCR, as structural differences displayed by the highly similar NAD and SFD are sufficient to hinder interaction with one or both proteins, thereby failing to trigger CTL activation. While HLA-B*13:01 has been previously known as a genetic marker for DDS-DIHS [32], our study also demonstrated that DDS-specific CTLs are characterized by TCRs of specific TRAV/TRBV and CDR3 clonotypes. Importantly, we found that these clonotypes can be detected in unstimulated PBMCs isolated directly from HLA-B*13:01-positive DDS-DIHS patients. This is in line with clinical observations and may explain why certain HLA-B*13:01 carriers are tolerant to DDS.

In addition, our in vitro co-culture assays with TCR transfectants further validated the pathogenic role of immunodominant TCR clonotype (i.e., TRAV12-3/TRBV28 pair and specific CDR3 region) in DDS-DIHS patients, and the CTLs expressing this TCR clonotype were demonstrated to produce high levels of inflammatory cytokines and cytotoxins in DDS- and HLA-B*13:01-dependent manner. Similarly, recent studies found shared TCR clonotypes in the blister cells [58] and in vitro expanded T cells [59] from PBMCs of carbamazepine-induced SJS/TEN patients. Apart from susceptible HLA alleles, the specific TCR clonotypes were deficient for drug metabolism/clearance that has also been linked to SCARs [15]. Thus, the risk factors for DDS-induced hypersensitivity syndrome include HLA-B*13:01 allele, specific TCR clonotypes, and impaired DDS clearance.

## Conclusions

In this study, we propose that the existence of both HLA-B*13:01 and T cells with specific TCR clonotypes are required to trigger immune responses in patients with DDS-induced hypersensitivity syndrome. The HLA-B*13:01-DDS-TCR immune synapse is formed according to the novel p-i HLA/TCR model, in which DDS directly interacts both with HLA-B*13:01 and the TCR with specific clonotype. These findings not only reveal the general molecular mechanism for HLA-restricted drug hypersensitivity reactions, but also provide a framework for prevention of DDS-DIHS and development of susceptibility gene diagnosis (detection of HLA-B*13:01 and TCR clonotype) and T cell immunotherapy (antibody therapy for TCR clonotype) for treatment of its symptoms.

## Supplementary Information

**Additional file 1.** Methods.

**Additional file 2: Figure S1.** Flow cytometry analysis of DDS-specific T cells in patients with DIHS. A We performed in vitro T cells expansion for day0, day21 and day35 of PBMC from DDS-induced DIHS patients and tolerant donors, as measured the ratios of CD8^+^ and CD4^+^ T cell by flow cytometry. B, C The ratios of CD8^+^ and CD4^+^ T cells of DDS-induced DIHS patients and tolerant individuals after T cells expansion were shown. The entire experiment was repeated thrice. *P < 0.05, **P < 0.01, ***P < 0.001, by two-tailed Student t test. **Figure S2.** The results of IFN-γ ELISpot assays. A DDS-specific CTLs activation was detected by IFN-γ ELISpot assays in a DDS-concentration manner. B T cells-mediated were blocked by anti–HLA class I antibody but not by anti-HLA class II antibody. **Figure S3.** Determination of the cross-reactivity of DDS and other sulfa drugs for dapsone-SCAR patients carried HLA-B*13:01. A Granulysin-based lymphocyte activation test (LAT) was performed in 6 dapsone-induced SCAR patients carried HLA-B*13:01 and 5 tolerant controls. A positive result was defined as a 1.3-fold increase in granulysin release compared to the controls (dashed blue line). Black solid dot, dapsone-SJS patient; blue solid dot, dapsone-DRESS patient, gray solid dot, dapsone-tolerant subject. B the sensitivity and specificity of LAT for dapsone-induced SCAR patients carried HLA-B*13:01 and their cross-reactive to dapsone are showed. **Figure S4.** SDS-PAGE analysis for β2M and HLA-B*13:01 protein. A 1. Purified HLA-B*13:01; 2. purified β2M; 3. Marker; 4. HLA-B*13:01-β2M complex. B the recombinant HLA-B*13:01 protein bounded to DDS in a DDS-concentration manner, with an estimated low-affinity of the micromolar range (Kd = 68.54 M). **Figure S5.** Pulsing assays and LC–MS analysis peptides involved in DDS binding with HLA-B*13:01. A Peptides were analyzed on C1R (HLA-B*13:01) with DDS cocultured by Co-IP and LC–MS. B T Lymphocyte activation test was performed from C1R (HLA-B*13:01) cultured with DDS or two peptides by IFN-γ release. **Figure S6.** Flow cytometry analysis of DDS-specific TCR transfectants. TRA12-3/TRB28, TCRA13-1/TRB5-1 and TCRA12-3/TRB30 were reconstituted and overexpressed into PBMC form DDS tolerant subjects, and the expression levels of TCRαβ and GFP were determined by FACS.

**Additional file 3: Table S1.** TCR primers used in RT-PCR analysis.

## Data Availability

The datasets supporting the conclusions of this article are included in this published article (and its Additional files).

## Abbreviations

ALDEN: Algorithm of drug causality for epidermal necrolysis
APC: Antigen presenting cell
BLCLs: B-cell lines
β2M: Beta-2-microglobulin
CBZ: Carbamazepine
CDR3: Third complementarity-determining region
CTLs: Cytotoxic T lymphocytes
DDS: Dapsone
DEGs: Differentially expressed genes
DIHS: Drug-induced hypersensitivity syndrome
DDS-DIHS: Dapsone-induced hypersensitivity syndrome
DMSO: Dimethyl sulfoxide
DRESS: Drug reaction with eosinophilia and systemic symptoms
DTH: Delayed-type hypersensitivity reactions
ELISA: Enzyme-linked immunosorbent assays
ELISPOT: Enzyme-linked immunospot
HLA: Human leukocyte antigens
LTG: Lamotrigine
MPE: Maculopapular exanthema
NAD: N-acetyl DDS
PBMC: Peripheral blood mononuclear cells
PHA: Phytohemagglutinin
p-i: Pharmacological interaction with immune receptors
SCAR: Severe cutaneous adverse reactions
SAD: Single-wavelength anomalous dispersion
SFD: Sulfadiazine
SJS: Stevens-Johnson syndrome
SSRF: Shanghai Synchrotron Radiation Facility
SPR: Surface plasmon resonance
TCE: Trichloroethylene
TCR: T cell receptor
TEN: Toxic epidermal necrolysis
TRAV genes: T cell receptor alpha variable genes
TRBV genes: T cell receptor beta variable genes
