# Supplementary material for: Functional and structural characteristics of HLA-B*13:01-mediated specific T cells reaction in dapsone-induced drug hypersensitivity
Source: J Biomed Sci. 2022 Aug 13;29:58. doi: 10.1186/s12929-022-00845-8 (PMC9375929; doi:10.1186/s12929-022-00845-8)
Supplement: Supplementary file 1 — Additional file 1. Methods. [file 12929_2022_845_MOESM1_ESM.docx]

**Supplementary methods**

**Homology modeling.** The amino acid sequence of HLA-B*13:02 was obtained from the NCBI database (<http://www.ncbi.nlm.nih.gov/protein/>) (BAA08822.1). A sequence similarity search was carried out with BLAST to identify the templates of them. The accuracy of the homology modeling structure was depending on the similarity between the model sequence and the templates. When the sequence similarity is above 25%, BLAST can effectively identify the correct templates. After aligning the model sequence with the selected templates, we built the three-dimensional models of HLA-B*13:02 sequences by the MODELER program in Accelrys Discovery Studio 3.0 (DS3.0) and all of the models are sorted by the PDF Total Energy. The model structures were further evaluated with the Verify Protein (Profiles-3D) protocol, which assesses the compatibility of the 3D structure of a protein model with the sequence of residues it contains. Furthermore, the Ramachandran Plot was generated to verify the predicted torsion angles of residues in modeling proteins.

**Detection of the ligand binding site.** The ligand binding site was detected by DS3.0 and Sitemap module in Schrödinger 2009. In DS3.0, the binding site was detected by checking the cavity of protein structure. In Sitemap module, the force field OPLS_2005 was used in the detection. The required site points per reported site were set as 15, and more restrictive definition of hydrophobicity was set. Standard grid was used as for the output 5 sites arranged by their Sitescore.

**Molecular docking****.** Molecular docking was applied to obtain the accurate binding mode of the HLA, peptide and DDS. Peptide docking was completed by ZDock with the force field CHARMm and MMFF94 charge method. The binding site for peptide binding was defined by that in the template protein. The output clusters and conformations were set as 10 and 200, respectively. CDocker and Glide were applied for ligand docking with the detected binding site for ligand binding used. DDS was prepared using Ligpre module in Schrödinger 2009. The protein structure was prepared using Protein Preparation Wizard. The extra precision mode was set for molecular docking.
