# Supplementary material for: Functional and structural characteristics of HLA-B*13:01-mediated specific T cells reaction in dapsone-induced drug hypersensitivity
Source: J Biomed Sci. 2022 Aug 13;29:58. doi: 10.1186/s12929-022-00845-8 (PMC9375929; doi:10.1186/s12929-022-00845-8)

Supplementary Figure 1

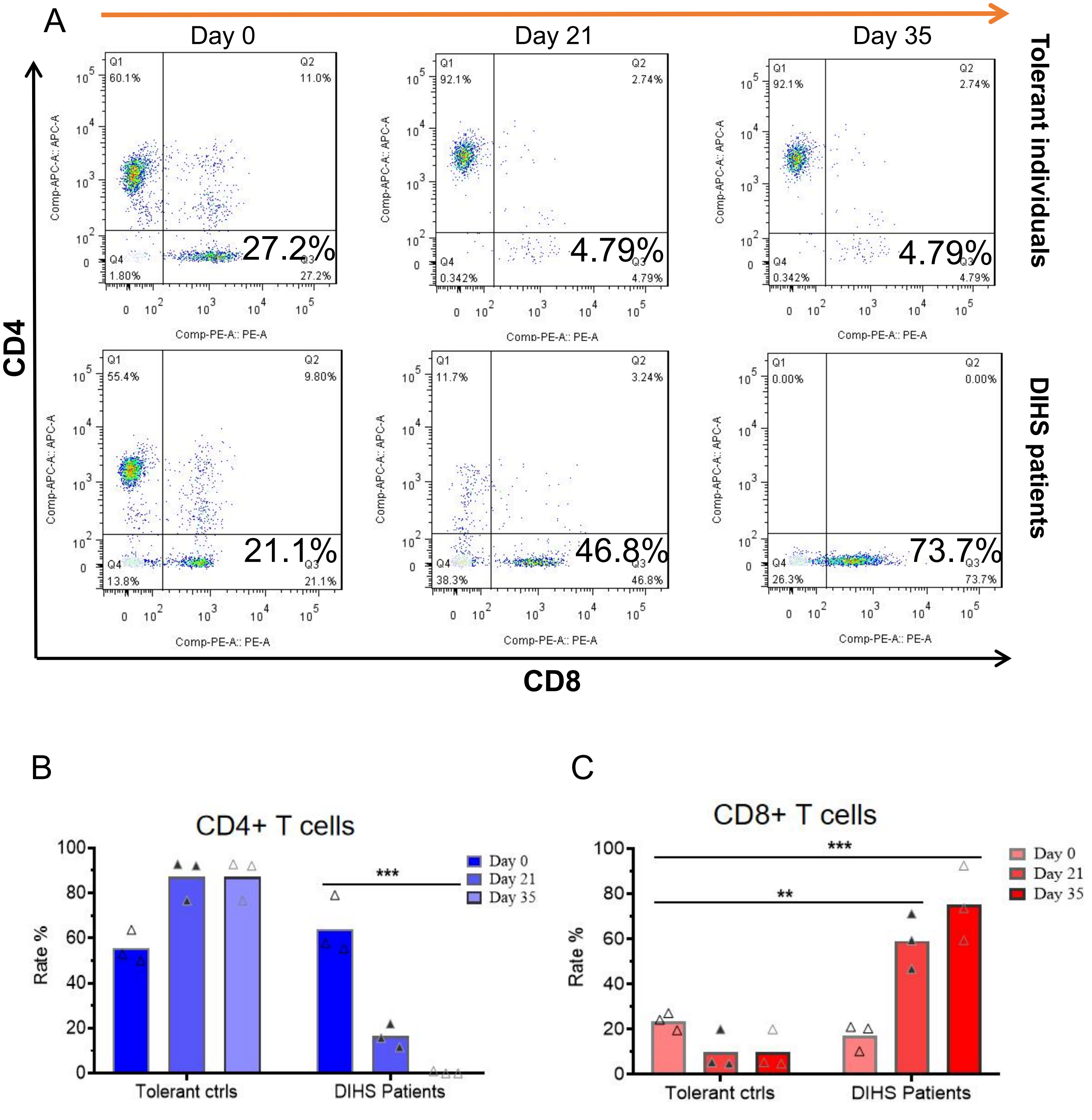

Supplementary Figure 2

A

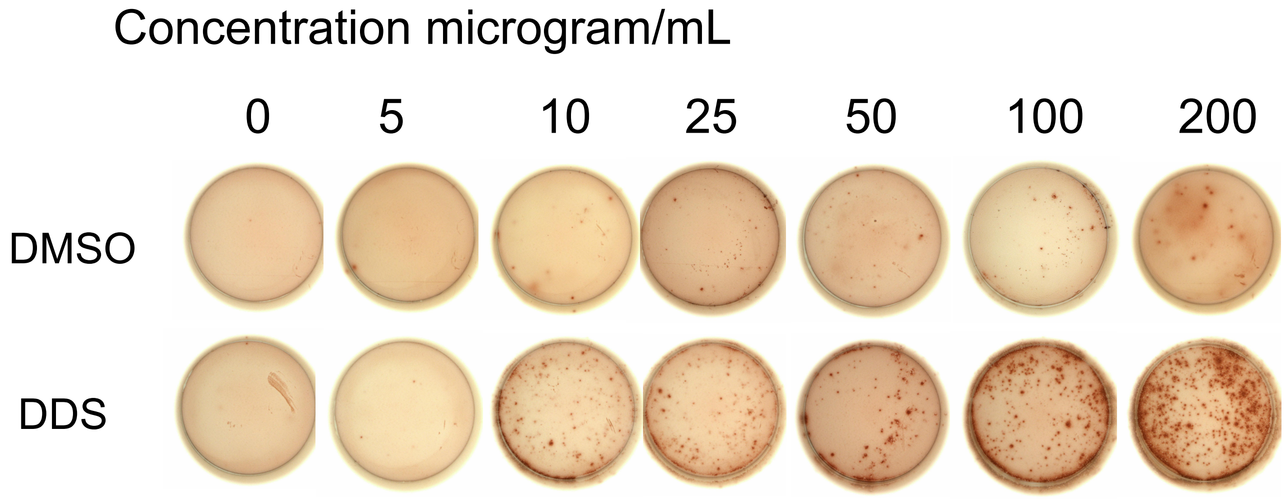

B

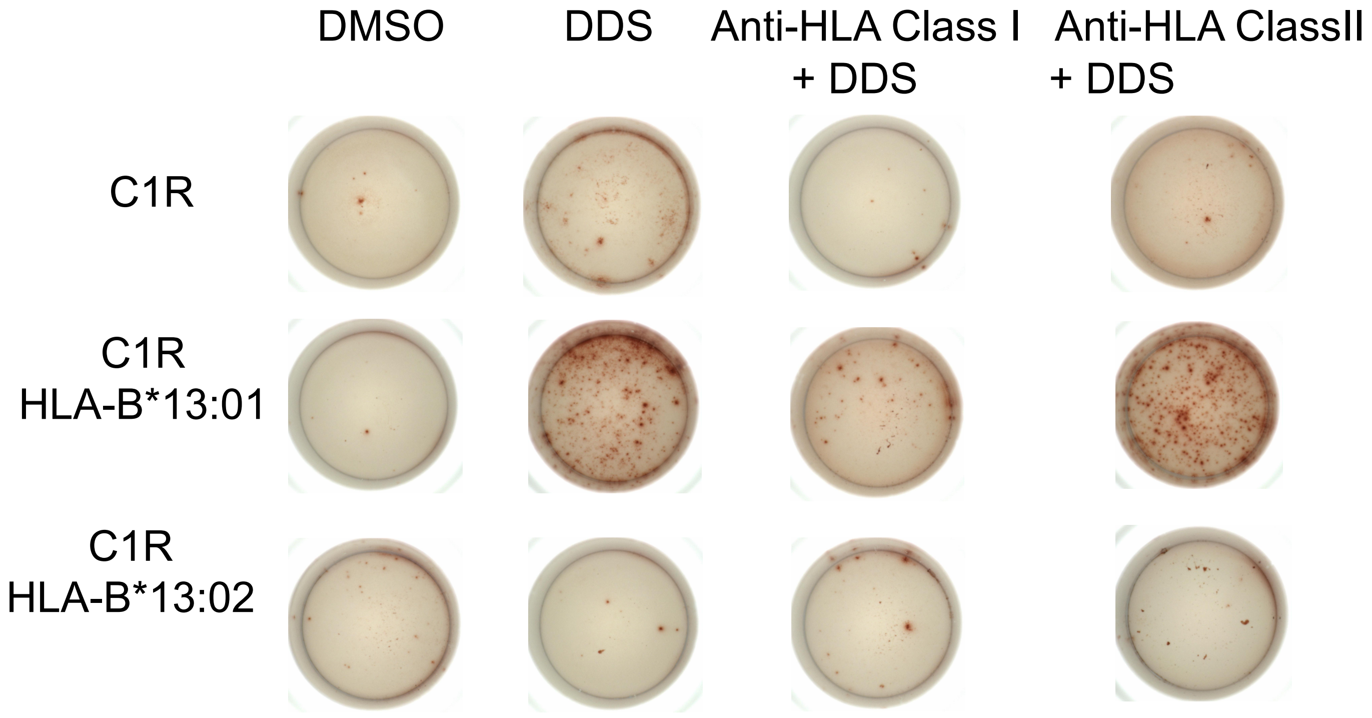

Supplementary Figure 3

A

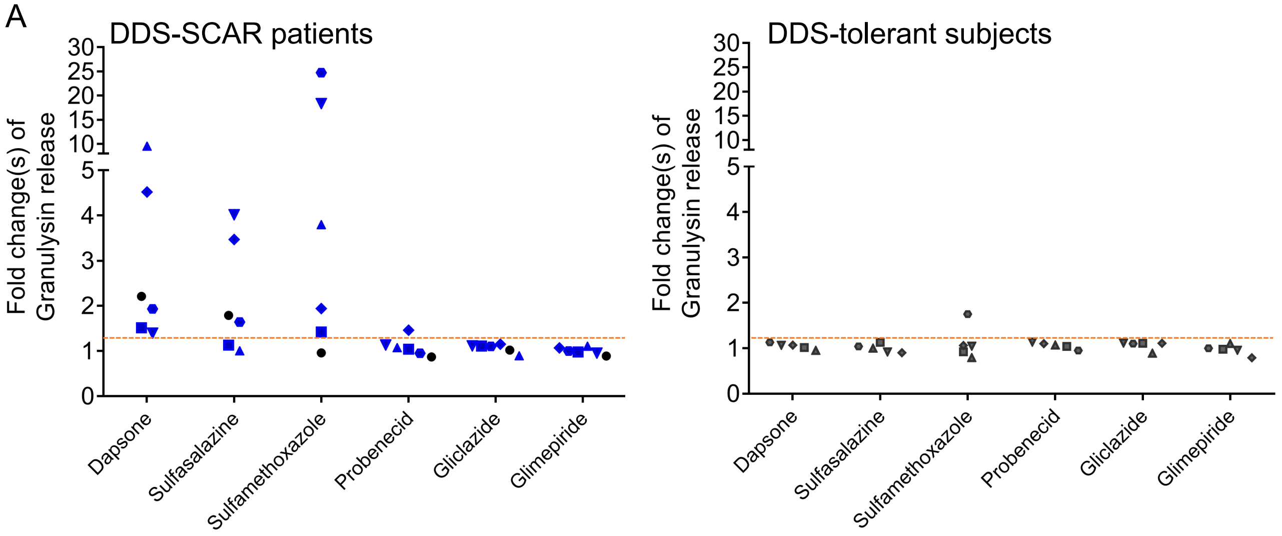

B

DDS-SCAR cases carried HLA-B*13:01	Dapsone	Sulfasalazine (cross-reactivity)	Sulfamethoxazole (cross-reactivity)	Tolerant control
**Positive LAT/ Cases**	6/6	4/6	5/6	0/5
**Sensitivity**	100%	66.7%	83.3%	/
**Specificity**	100%	100%	83.3%	/

Supplementary Figure 4

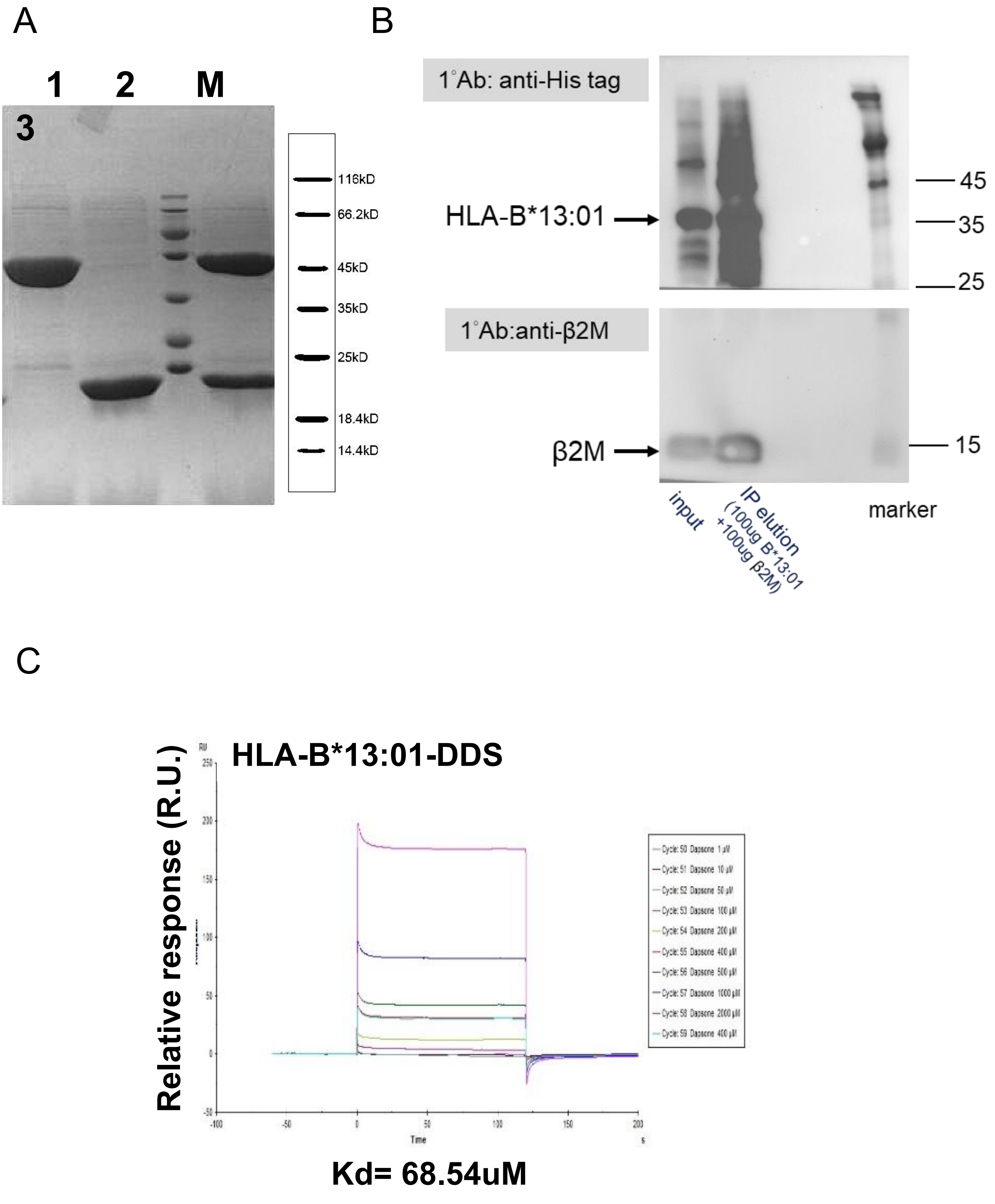

Supplementary Figure 5

A

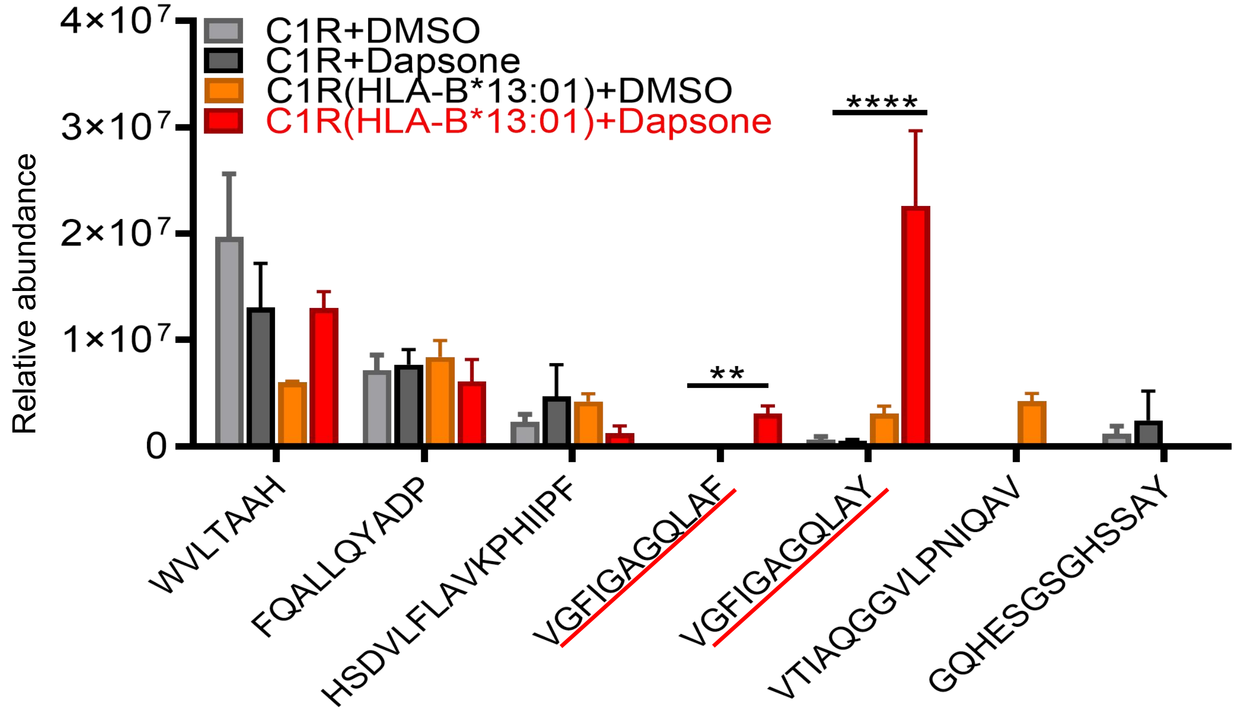

B

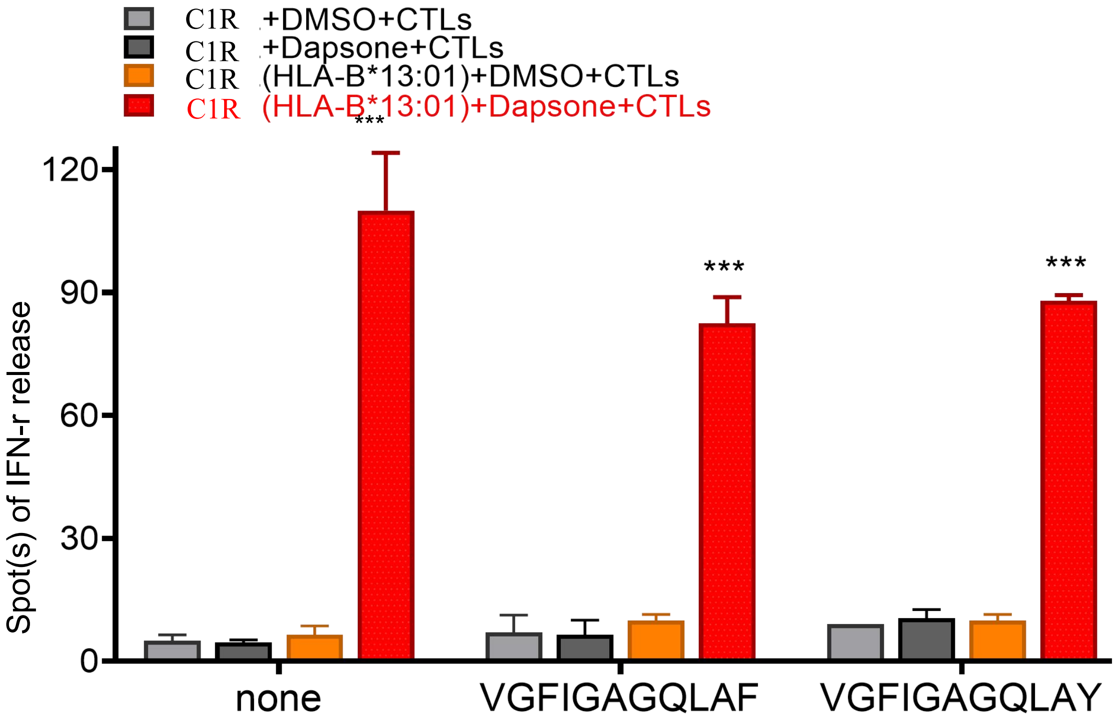

Supplementary Figure 6

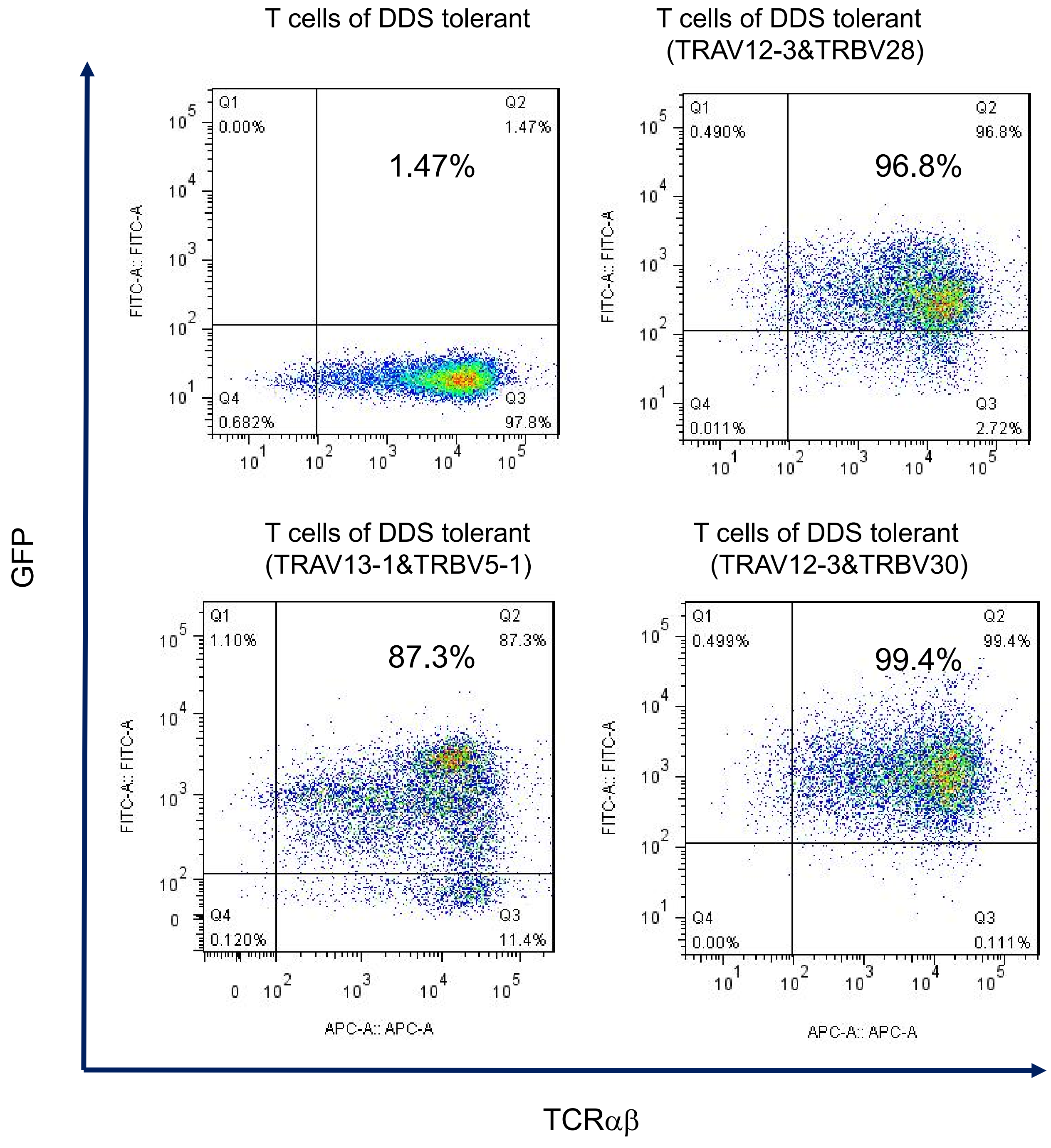

Supplement: Supplementary file 2 — Additional file 2: Figure S1. Flow cytometry analysis of DDS-specific T cells in patients with DIHS. A We performed in vitro T cells expansion for day0, day21 and day35 of PBMC from DDS-induced DIHS patients and tolerant donors, as measured the ratios of CD8+ and CD4+ T cell by flow cytometry. B, C The ratios of CD8+ and CD4+ T cells of DDS-induced DIHS patients and tolerant individuals after T cells expansion were shown. The entire experiment was repeated thrice. *P < 0.05, **P < 0.01, ***P < 0.001, by two-tailed Student t test. Figure S2. The results of IFN-γ ELISpot assays. A DDS-specific CTLs activation was detected by IFN-γ ELISpot assays in a DDS-concentration manner. B T cells-mediated were blocked by anti–HLA class I antibody but not by anti-HLA class II antibody. Figure S3. Determination of the cross-reactivity of DDS and other sulfa drugs for dapsone-SCAR patients carried HLA-B*13:01. A Granulysin-based lymphocyte activation test (LAT) was performed in 6 dapsone-induced SCAR patients carried HLA-B*13:01 and 5 tolerant controls. A positive result was defined as a 1.3-fold increase in granulysin release compared to the controls (dashed blue line). Black solid dot, dapsone-SJS patient; blue solid dot, dapsone-DRESS patient, gray solid dot, dapsone-tolerant subject. B the sensitivity and specificity of LAT for dapsone-induced SCAR patients carried HLA-B*13:01 and their cross-reactive to dapsone are showed. Figure S4. SDS-PAGE analysis for β2M and HLA-B*13:01 protein. A 1. Purified HLA-B*13:01; 2. purified β2M; 3. Marker; 4. HLA-B*13:01-β2M complex. B the recombinant HLA-B*13:01 protein bounded to DDS in a DDS-concentration manner, with an estimated low-affinity of the micromolar range (Kd = 68.54 M). Figure S5. Pulsing assays and LC–MS analysis peptides involved in DDS binding with HLA-B*13:01. A Peptides were analyzed on C1R (HLA-B*13:01) with DDS cocultured by Co-IP and LC–MS. B T Lymphocyte activation test was performed from C1R (HLA-B*13:01) cultured with [file 12929_2022_845_MOESM2_ESM.pdf]
