# Supplementary material for: Functional and structural characteristics of HLA-B*13:01-mediated specific T cells reaction in dapsone-induced drug hypersensitivity
Source: J Biomed Sci. 2022 Aug 13;29:58. doi: 10.1186/s12929-022-00845-8 (PMC9375929; doi:10.1186/s12929-022-00845-8)
Supplement: Supplementary file 3 — Additional file 3: Table S1. TCR primers used in RT-PCR analysis. [file 12929_2022_845_MOESM3_ESM.pdf]

Supplementary Table 1 TCR primers used in RT-PCR analysis

Primers	Sequence (5'-3')	Amplified gene
TRAV12-3 Forward	TCAGACGTTCCCTGTGATGC	TRAV12-3
TRAV12-3 Reverse	TGTTTCACGCCAGAACCACT	
TRAV13-1 Forward	ACAGCGCCTTCCAGTACTTC	TRAV13-1
TRAV13-1 Reverse	GTCGGAAGGCTGGCTATCTC	
TRBV28 Forward	GGCTCTCGGAGAATGACGAG	TRBV28
TRBV28 Reverse	GACACCGAGGTAAAGCCACA	
TRBV30 Forward	GGCTCTCGGAGAATGACGAG	TRBV30
TRBV30 Reverse	GACACCGAGGTAAAGCCACA	
GAPDH Forward	CGACCACTTTGTCAAGCTCA	GAPDH
GAPDH Reverse	AGGGGTCTACATGGCAACTG	
